# Environmental volunteer well-being: Managers’ perception and actual well-being of volunteers

**DOI:** 10.12688/f1000research.10016.1

**Published:** 2016-11-16

**Authors:** Gitte Kragh, Rick Stafford, Susanna Curtin, Anita Diaz

**Affiliations:** 1 Department of Life & Environmental Sciences, Faculty of Science and Technology, Bournemouth University, Poole, UK; 2 Department of Tourism and Hospitality, Faculty of Management, Bournemouth University, Poole, UK

**Keywords:** citizen science, environmental volunteering, nature-based activities, PERMA, positive psychology, practical conservation, volunteering, well-being

## Abstract

*Background*: Environmental volunteering can increase well-being, but environmental volunteer well-being has rarely been compared to participant well-being associated with other types of volunteering or nature-based activities. This paper aims to use a multidimensional approach to well-being to explore the immediately experienced and later remembered well-being of environmental volunteers and to compare this to the increased well-being of participants in other types of nature-based activities and volunteering. Furthermore, it aims to compare volunteer managers’ perceptions of their volunteers’ well-being with the self-reported well-being of the volunteers. *Methods*: Onsite surveys were conducted of practical conservation and biodiversity monitoring volunteers, as well as their control groups (walkers and fieldwork students, respectively), to measure general well-being before their nature-based activity and activity-related well-being immediately after their activity. Online surveys of current, former and potential volunteers and volunteer managers measured remembered volunteering-related well-being and managers’ perceptions of their volunteers’ well-being. Data were analysed based on Seligman’s multidimensional PERMA (‘positive emotion’, ‘engagement’, ‘positive relationship’, ‘meaning’, ‘achievement’) model of well-being. Factor analysis recovered three of the five PERMA elements, ‘engagement’, ‘relationship’ and ‘meaning’, as well as ‘negative emotion’ and ‘health’ as factors. *Results*: Environmental volunteering significantly improved positive elements and significantly decreased negative elements of participants’ immediate well-being, and it did so more than walking or student fieldwork. Even remembering their volunteering up to six months later, volunteers rated their volunteering-related well-being higher than volunteers rated their well-being generally in life. However, volunteering was not found to have an effect on overall mean well-being generally in life. Volunteer managers did not perceive the significant increase in well-being that volunteers reported. *Conclusions*: This study showed how environmental volunteering immediately improved participants’ well-being, even more than other nature-based activities. It highlights the benefit of regarding well-being as a multidimensional construct to more systematically understand, support and enhance volunteer well-being.

## Introduction

Natural environments have always been important for human well-being ( Frumkin, 2001; Kellert & Wilson, 1993), and continue to be so as local environments become more urbanised ( Kaplan, 1983). One way to harness the well-being benefits of natural environments is to participate in environmental volunteering, which can increase people’s connection to nature and their sense of well-being ( Gooch, 2005; O’Brien *et al.*, 2010; Pillemer *et al.*, 2010). Most research on volunteer well-being has focused on comparisons between volunteers and non-volunteers, elucidating differences in specific elements of well-being, such as happiness, life satisfaction, depression and survival ( Jenkinson *et al.*, 2013; Konrath *et al.*, 2012; Thoits & Hewitt, 2001). Very few studies have addressed the questions of how volunteering immediately affects participants’ well-being and how participants in different types of volunteering may gain benefits in different elements of well-being. In addition, no studies have examined how volunteer managers perceive the well-being of their volunteers and how this relates to actual volunteer well-being. This paper addresses these challenges by using a multidimensional well-being model to first explore the well-being of environmental volunteers and compare it to the well-being of participants in other similar types of nature-based activities and other types of volunteering. It then explores volunteer managers’ perception of the well-being of their volunteers, and finally it compares this perceived well-being to the volunteers’ self-reported well-being.

### Volunteer well-being

Many studies have shown that volunteering is closely linked to increased well-being of volunteers ( Binder & Freytag, 2013; Borgonovi, 2008; Greenfield & Marks, 2004; Jenkinson *et al.*, 2013; Koss & Kingsley, 2010; O’Brien *et al.*, 2010; Son & Wilson, 2012; Stukas *et al.*, 2016; Thoits & Hewitt, 2001; Townsend, 2006; Van Willigen, 2000; Wheeler *et al.*, 1998; Wilson, 2000). However, studies have used different definitions of well-being, and have therefore measured different constructs, which have often included only some aspects of well-being instead of taking a holistic approach. Two main approaches to conceptualising well-being prevail: hedonism and eudaimonia. Hedonism is the idea that maximisation of pleasure is the goal and the way to happiness for all humans, whereas eudaimonia proposes that striving to lead a meaningful life and achieve optimum functioning is the way to happiness ( Aristotle, 2009; Diener, 2000; Ryan & Deci, 2001; Ryff, 1989). The two approaches have informed research into human well-being with different methods proposed for the study of well-being. Methods based on the study of ‘subjective well-being’ includes measures of positive affect, negative affect and life satisfaction, a mixture of both hedonic and eudaimonic well-being ( Bradburn, 1969; Diener, 1984; Diener, 1994; Diener *et al.*, 1999). The study of ‘psychological well-being’ on the other hand measures only eudaimonic elements of life, such as self-acceptance, positive relations with others, autonomy, environmental mastery, purpose in life and personal growth, leaving out the hedonic focus on pleasures ( Ryff, 1989; Ryff, 1995; Ryff, 2014).

Though some aspects of volunteer well-being have been studied in depth, no previous studies have investigated volunteer managers’ perceptions of the well-being of their volunteers. As volunteer managers are responsible for the well-being of their volunteers, and as improved volunteer well-being is often an important outcome for volunteers, organisations and society ( O’Brien *et al.*, 2011), it is vital that managers’ perceptions of the well-being of their volunteers correspond to actual volunteer well-being. The cumulative evidence from a broad range of studies (see meta-analyses and reviews in Jenkinson *et al.*, 2013; Musick & Wilson, 2008; Wilson, 2000; Wheeler *et al.*, 1998) is that volunteering has a positive relationship with a wide range of elements within the concept of well-being, though causation can be difficult to determine ( Greenfield & Marks, 2004). Previous studies have investigated the effect of volunteering on subjective well-being (e.g. Binder & Freytag, 2013; Harlow & Cantor, 1996; Windsor *et al.*, 2008) or psychological well-being (e.g. Ho, 2015), or a combination of one of these along with other elements of well-being, such as social well-being, trust, self-esteem, depression or physical health (e.g. Greenfield & Marks, 2004; Koss & Kingsley, 2010; O’Brien *et al.*, 2010; Son & Wilson, 2012; Stukas *et al.*, 2016; Thoits & Hewitt, 2001; Townsend, 2006). Some studies show that volunteering leads to increased well-being ( Borgonovi, 2008; Piliavin, 2009; Piliavin & Siegl, 2007), while other studies show that people higher in well-being are also more likely to volunteer ( Gimenez-Nadal & Molina, 2015; Greenfield & Marks, 2004) and to volunteer more hours ( Son & Wilson, 2012; Thoits & Hewitt, 2001). Most likely the causality runs both ways between volunteering and well-being ( Binder & Freytag, 2013; Gimenez-Nadal & Molina, 2015) in a ‘virtuous cycle’ where happy and healthy people volunteer more and volunteers are happier and healthier ( Brooks, 2007). Environmental volunteering could further enhance this virtuous cycle, as spending time in nature has been linked to increased well-being ( Frumkin, 2001).

### Environmental volunteer well-being

Only a few studies have focused specifically on the relationship between environmental volunteers and their well-being (e.g. Koss & Kingsley, 2010; O’Brien *et al.*, 2010; Townsend, 2006), as many studies have used cohort datasets where volunteering type was often heterogeneous or not described ( Jenkinson *et al.*, 2013). Volunteering in nature has been linked to well-being benefits for volunteers, including improved social networks ( Bell *et al.*, 2008; Gooch, 2005; Koss & Kingsley, 2010; Muirhead, 2011; O’Brien *et al.*, 2010), increased personal satisfaction and feelings of enjoyment ( Koss & Kingsley, 2010; Muirhead, 2011), and improved health and well-being ( Koss & Kingsley, 2010; O’Brien *et al.*, 2010; Pillemer *et al.*, 2010). Environmental volunteering can have a positive effect, not only by increasing positive indices of well-being, but also by reducing negative indices such as reducing stress ( Guiney & Oberhauser, 2009; O’Brien *et al.*, 2010) and depression ( Pillemer *et al.*, 2010). Furthermore, environmental volunteering offers the added benefit of providing opportunities for volunteers to spend time in nature, which can lead to a better connection or re-connection with nature for the volunteers ( Bell *et al.*, 2008; Guiney & Oberhauser, 2009). It can also lead to volunteers gaining an increased understanding of the natural environment ( Koss & Kingsley, 2010) and thereby also an enhanced sense of place ( Evans *et al.*, 2005; Gooch, 2005). A closer connection to nature has been shown to enhance people’s well-being ( Bowler *et al.*, 2010; Kellert & Wilson, 1993), and therefore it could be expected that environmental volunteers would benefit more from their volunteering than other types of volunteers. Practical conservation volunteering requires stamina and physical strength and it provides a way to exercise and gain improved fitness ( Guiney & Oberhauser, 2009; O’Brien *et al.*, 2010), which can also reinforce positive well-being ( Pretty *et al.*, 2005).

To better understand these relationships between volunteering and well-being, a more holistic and multidimensional approach to well-being, including both hedonic and eudaimonic elements, as well as social elements, would be well suited ( Piliavin, 2009). Such a holistic approach to well-being is gaining acceptance ( Forgeard *et al.*, 2011; Keyes, 2002; Ryan & Deci, 2001), and one proposed multidimensional model of well-being is Seligman’s (2011) PERMA model. It is a construct with five contributing elements (PERMA): 1) ‘Positive emotion’, which encompass present positive feelings, life satisfaction and positive emotions about the future; 2) ‘engagement’, which is employing one’s strengths to a task, becoming fully absorbed in the task and therefore completely losing track of time, also referred to as getting into ‘flow’ ( Csikszentmihalyi, 1975; Csikszentmihalyi, 1991; Seligman, 2011); 3) ‘positive relationships’, which are fundamental to a good life according to Seligman (2011), and Baumeister & Leary (1995) have also defined it as a basic human need that is essential for well-being; 4) ‘meaning’, which includes feelings of doing something worthwhile and having a purpose and direction in life, something which is crucial to well-being as, according to Seligman (2011), most people have a need to belong to or serve something they believe is larger than themselves, e.g. their family, an organisation or a religious group; and 5) ‘achievement’, often pursued for its own sake by individuals setting their own personal goals or striving to achieve recognition in the wider world, e.g. winning an award or accumulating wealth. Seligman (2011) did not propose a measure for his PERMA model but Butler & Kern (2016) subsequently developed the PERMA-Profiler (PERMA-P), a scale based on the PERMA model, which also includes additional elements of well-being. The additional elements in the PERMA-P are 1) ‘negative emotion’ from the concept of subjective well-being acknowledging the importance of both positive and negative aspects of well-being; 2) ‘health’, which can be considered a core part of well-being; 3) ‘loneliness’, which is a strong predictor of many negative life outcomes; and 4) ‘overall happiness’, which allows an overall assessment after reflecting on specific elements of well-being ( Butler & Kern, 2016).

### Aims and research questions

This paper aims to use a multidimensional approach to well-being to explore the immediately experienced and later remembered well-being of environmental volunteers, as well as their general well-being and to compare this to the well-being of participants in other types of nature-based activities and volunteering. It also aims to compare volunteer managers’ perception of their volunteers’ well-being with the self-reported well-being of the volunteers. These aims were addressed through the following research questions: 1) How does environmental volunteering immediately affect participants’ sense of well-being, and how does that compare to the immediate effect of other types of nature-based activities on participants’ sense of well-being? 2) How well do volunteers sustain the memory of this immediately experienced sense of well-being after they have gone home? 3) How do volunteer managers perceive the effect of volunteering on the well-being of their volunteers? 4) How does the volunteer managers’ perception of volunteer well-being compare to volunteers’ actual sense of volunteering-related well-being?

## Methods

Well-being was investigated using a positive psychology approach based on the PERMA well-being theory proposed by Seligman (2011) and using the PERMA-Profiler (PERMA-P) developed by Butler & Kern (2016). The PERMA-P consists of the original five well-being elements proposed by Seligman, ‘positive emotion’ (P), ‘engagement’ (E), ‘positive relationships’ (R), ‘meaning’ (M) and ‘achievement’ (A), as well as ‘negative emotion’ and ‘health’, measured with three items each, and ‘loneliness’ and ‘happiness’, measured with a single item each. Three-item elements can be regarded as individual factors or elements, and the resulting PERMA-P seven-factor model of well-being can be tested through factor analysis with the ‘overall happiness’ and ‘loneliness’ items providing additional information ( Butler & Kern, 2016). All items were scored on an 11-point (0–10) Likert scale ( Likert, 1932). Following a pilot study (unpublished report, GK, RS, SC and AD), the wording of two items on the questionnaire was changed. The two words, ‘loved’ and ‘angry’, were seen by volunteers to be ‘quite American’ and badly fitted to a British volunteering context, and were therefore changed to ‘appreciated’ and ‘frustrated’, respectively. Data presented here are the complete subset of all items related to well-being in the questionnaires from a larger study, which also investigated volunteer motivation and activities (GK PhD research). Data were obtained from three sources: Study 1) an onsite survey of participants in nature-based activities ( Dataset 1); Study 2) an online survey of former, current and potential volunteers ( Dataset 2); and Study 3) an online survey of former and current volunteer managers ( Dataset 3; Table 1). 

**Table 1.** Overview of the three studies, respondents and type of well-being measured. Overview of the three studies in this research, including focus, respondents, subgroups and type of well-being measured. BM, biodiversity monitoring volunteers; Stud, Students conducting fieldwork as part of their university course; PC, practical conservation volunteers; Walk, walkers; BMPC, biodiversity monitoring volunteers also doing practical conservation.

	Study 1: Onsite activity survey							
**Respondents**	Activity participants (volunteers, students and walkers)							
**Focus**	Before-activity				After-activity			
**Type of well-being** **measured**	Own general well-being				Own experienced activity-related well-being			
**Respondent sub-** **groups**	BM	Stud	PC	Walk	BM	Stud	PC	Walk
	**Study 2: Online volunteer survey**							
**Respondents**	Volunteers							
**Focus**	Current				Former and potential			
**Type of well-being** **measured**	Own remembered activity- related well-being				Own general well-being			
**Respondent sub-** **groups**	BM	BMPC	PC	Other	BM	BMPC	PC	Other
	**Study 3: Online volunteer manager survey**							
**Respondents**			Volunteer managers					
**Focus**			Former and current					
**Type of well-being** **measured**			Perceived volunteer well-being					
**Respondent sub-** **groups**	BM		BMPC		PC		Other	

The aim of Study 1 was to answer research question 1) How does environmental volunteering immediately affect participants’ sense of well-being and how does that compare to the immediate effect of other types of nature-based activities on participants’ sense of well-being? Combining data from Study 1 and Study 2 aimed to answer research question 2) How well do volunteers sustain the memory of this immediately experienced sense of well-being after they have gone home? The aim of Study 3 was to answer research question 3) How do volunteer managers perceive the effect of volunteering on the well-being of their volunteers? And finally, combining data from all three studies aimed to answer research question 4) How does this volunteer manager perception of volunteer well-being compare to volunteers’ actual sense of volunteering-related well-being?

### Participants

***Ethics.*** This research project was approved through the ethics approval process at Bournemouth University (ref ID 2419). All participants provided written informed consent for participation.

***Study 1.*** The onsite study was conducted between October 2014 and November 2015 and involved participants from 13 organisations from Southern England, divided into four types of activities: Biodiversity monitoring, practical conservation volunteering, walking, and students conducting fieldwork as part of their university course ( Table 2). Environmental organisations were invited to participate in the study based on them conducting volunteer activities in groups. Control groups were invited based on their group activity being conducted in the same natural environments as the volunteer activities of the environmental organisations. To determine if environmental volunteering had a different effect on well-being compared to other non-altruistic activities performed outdoors, students and walkers were surveyed in addition to environmental volunteers. Students were chosen as the control group to the biodiversity monitoring volunteers, as both groups were conducting ecological fieldwork in similar areas, but whereas volunteering is often seen as altruistic ( Smith, 1981; Unger, 1991), students did the fieldwork because it was a requirement of their university courses. Walking groups were chosen as the control group for the practical conservation volunteers as both activities were performed outdoors in similar areas and were somewhat physically demanding, but the purpose of the activities were again different, with volunteering being partly altruistic and walking only benefitting the walkers themselves. Also, walking is the most popular activity in the natural environment in England ( Natural England, 2015) and walking programmes are promoted as health interventions to decrease negative affect and mental illness and increase well-being in participants ( Iwata *et al.*, 2016; Marselle *et al.*, 2014). The survey was designed as a paired before-activity and after-activity survey to measure general level of well-being and experienced level of well-being during an activity, respectively. Activity participants only completed questionnaires once to ensure independent samples even if they participated in activities later where other activity participants completed questionnaires.

**Table 2.** Respondents and descriptive statistics of groups in the onsite survey (Study 1).

Activity type	n _general well-being_	n _activity well-being_	Number of organisations	Number of sample dates	Group sizes (mean ±SD)	Hours of activity (mean ±SD)
Biodiversity monitoring	91	79	8	16	12.83 *(±6.16)*	3.71 *(±1.62)*
Students	123	109	3	6	39.20 *(±21.72)*	3.95 *(±1.20)*
Practical conservation	100	101	2	15	15.62 *(±9.52)*	4.57 *(±1.06)*
Walkers	73	62	2	10	23.70 *(±4.28)*	2.77 *(±0.79)*

***Studies 2 and 3.*** Both online surveys were open to anyone with the link between September and December 2015. Environmental organisations involved in study 1 as well as other worldwide environmental organisations and volunteer centres in the UK were contacted directly and asked to invite their volunteers and volunteer managers to participate and the surveys were also sent out more widely through professional networks. Study 2 investigated the general level of well-being of former and potential volunteers as well as the remembered level of well-being during volunteering of current volunteers. In Study 2, a total of 417 responses were received with completed questions about well-being. This sample comprised 53% females and 47% males. Age ranged from 18 to 94 years old (mean=54.86, *SD*=16.10). Most respondents had at least one university degree (65.23%) and many were retired (48.68%), some were in full-time (21.10%) or part-time (13.19%) employment and few were students (6.95%), not currently employed (5.28%) or homemakers (1.20%). Respondents were from 11 different countries, with the majority residing in the United Kingdom (88.49%). They named 118 different organisations they previously or currently volunteer for or would like to volunteer for in the future. Respondents included people from three different periods: former volunteers (18%), current volunteers (70%) and potential future volunteers (12%). They were grouped into four types of volunteers: biodiversity monitoring volunteers (BM; 21%), practical conservation volunteers (PC; 34%), biodiversity monitoring volunteers also performing practical conservation work (BMPC; 25%), and all other types of volunteers (19%) ( Table 3).

**Table 3.** Type of volunteers and volunteer status of respondents to the online volunteer survey (Study 2). BMPC, biodiversity monitoring volunteers also performing practical conservation work (n=417).

Volunteer type	Former volunteers (%)	Current volunteers (%)	Potential volunteers (%)	*Total (%)*
Biodiversity monitoring	4.08	15.35	1.20	20.62
BMPC	3.84	17.27	4.32	25.42
Practical conservation volunteers	6.00	24.94	2.88	33.81
Other types of volunteers	4.08	12.47	2.40	18.94
Undisclosed			1.20	1.20
*Total*	*17.99*	*70.02*	*11.99*	*100.00*

Study 3 investigated the perceived level of well-being of volunteers by former and current volunteer managers. A total of 96 responses were received with completed questions about well-being. This sample comprised 61% females and 39% males. Age ranged from 19 to 74 years old (mean=43.01, *SD*=13.03). Most respondents had at least one university degree (80%) and most respondents were in full-time (69%) or part-time (13%) employment, few were retired (10%), students (2%), not currently employed (1%) or homemakers (1%). Respondents were from 10 different countries, with the majority residing in the United Kingdom (80%). Respondents included people from two different periods: former volunteer managers (14%) and current volunteer managers (86%), and they identified 62 different organisations they previously or currently manage volunteers for. They were grouped into four types of volunteering similarly to the volunteers in Study 2: BM (20%), PC (26%), BMPC (35%) and all other types of volunteering (19%) ( Table 4).

**Table 4.** Type of volunteering and volunteer manager status of respondents (Study 3). BMPC, volunteer managers in biodiversity monitoring also performing practical conservation work (n=96).

Types of volunteering	Former managers (%)	Current managers (%)	*Total (%)*
Practical conservation	2.08	23.96	*26.04*
BMPC	9.38	26.04	*35.42*
Biodiversity monitoring		19.79	*19.79*
Other types of volunteering	2.08	16.67	*18.75*
*Total*	*13.54*	*86.46*	*100.00*

### Data analyses

***Deriving the well-being factors.*** The first step in exploring well-being was to test if the structures of self-reported well-being and managers’ perception of volunteer well-being were consistent with the proposed seven-factor PERMA-Profiler (PERMA-P) model ( Butler & Kern, 2016). This was done by performing exploratory factor analysis (EFA) on a subsample of self-reported well-being data to generate a best fit model. The generated model and the original seven-factor PERMA-P model were subsequently tested for best fit through confirmatory factor analysis (CFA) using the other subsample of collected data from participants, and the total combined sample. EFA was also performed on the volunteer manager data sample to generate a best fit model and confirmatory factor analysis was run on the generated model, the model generated from the self-reported subsample and the original seven-factor PERMA-P model to determine the best fit model.

**Figure 1.**
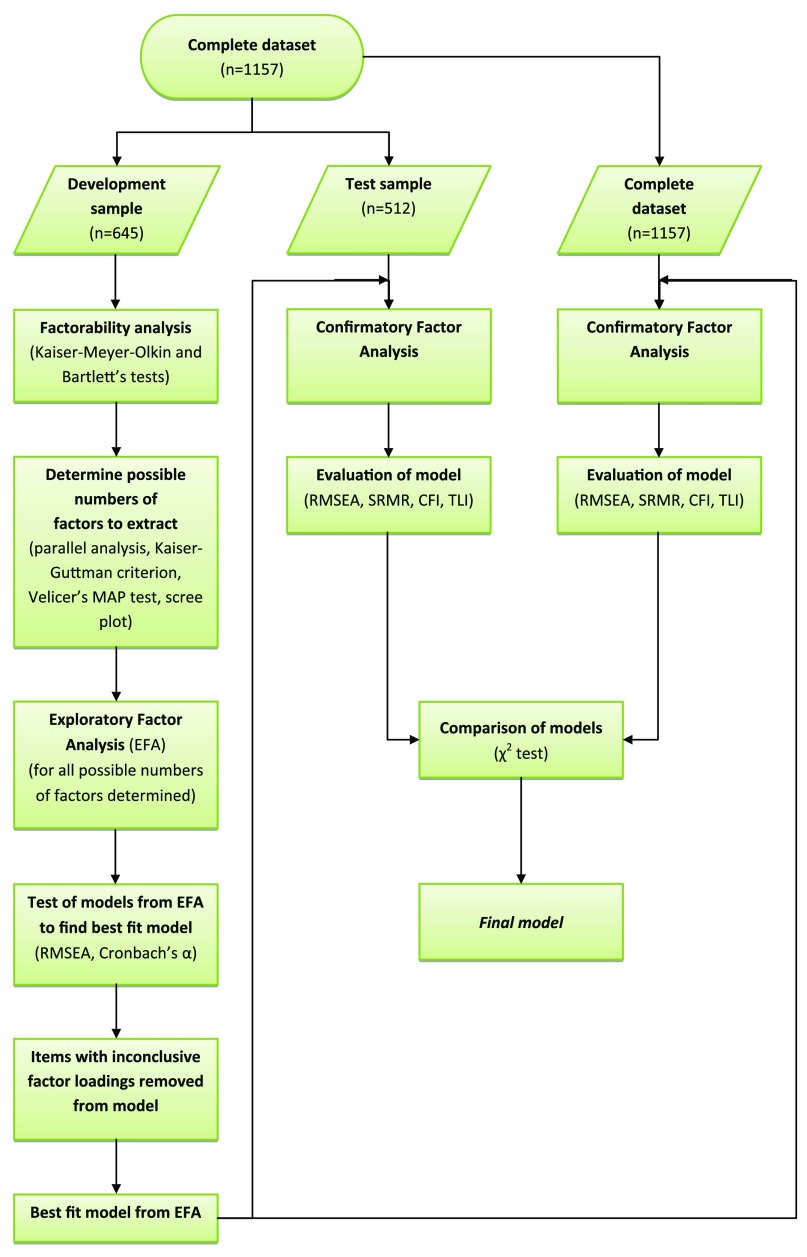
Analysis flowchart for determining the best fit model for self-reported well-being factors.

**Self-reported well-being:** Only complete responses were used for factor analysis (n=1157) ( Figure 1). The data were split in two subsamples to develop (n=645) and test (n=512) the factor model. The development sample consisted of all onsite and online respondents to questionnaires measuring activity-related well-being, which included volunteers and control activity participants from Study 1 (‘after-activity survey’) and current volunteers from Study 2. The test sample consisted of all onsite and online respondents to questionnaires measuring general well-being which included volunteers and control activity participants from Study 1 (‘before-activity survey’) and former and potential volunteers from Study 2. The largest subsample was used as the development sample for the EFA.

The first step in determining the best fitting model was to test the factorability of the items in the development subsample with the Kaiser-Meyer-Olkin measure of sampling adequacy, recommended to be >0.60, and with Bartlett’s test of sphericity, where significance indicates the data are suitable for factor analysis ( Dziuban & Shirkey, 1974). The first step in EFA is to determine the number of factors to extract. There is no set formula for determining this number and it is determined by using a variety of methods and interpretation of the data ( Matsunaga, 2010). Several methods were used to determine the number of factors to extract, including parallel analysis ( Horn, 1965), the Kaiser-Guttman criterion (counting only Eigenvalues above one, Kaiser, 1960), Velicer’s minimum average partial (MAP) test ( Velicer, 1976) and visual inspection of the scree plot ( Cattell, 1966). EFA using ordinary least squares to find the minimum residual (minres) solution with oblique (promax) rotation, which allows factors to be correlated, were performed for relevant models. To determine overall best fit model, results were evaluated using the root mean square error of approximation (RMSEA). RMSEA <0.05 indicate a good fit and between 0.05 and 0.08 indicate a fair fit ( MacCallum *et al.*, 1996). Cronbach’s α ( Cronbach, 1951) was calculated for each factor to test internal reliability of factors. Cronbach’s α values >0.70 are considered acceptable ( Nunnally, 1978), though for scales with 6 or fewer items lower α values may be acceptable ( Cortina, 1993). Items with factor loadings <0.04 or loading on two factors with the difference between primary and secondary loadings <0.03 were removed from the dataset before further analyses, a suggested way of dealing with inconclusive factor loadings ( Matsunaga, 2010). The best factor model was determined by choosing the model with optimal model fit indices, high internal reliability of factors and best interpretability of the data. CFA is a method to test if a certain predetermined model is a good fit for a data sample. CFA was performed for the best fit model developed from the EFA, the original seven-factor PERMA-P model and a generic one-dimensional control model using the test sample and the combined development and test sample. Model fits were evaluated using RMSEA, the standardised root mean residual (SRMR), comparative fit index (CFI) and the Tucker Lewis Index (TLI), and models were compared for best fit using χ ^2^ difference tests. SRMR below 0.08 is considered a good fit, and TLI and CFI values >0.90 are considered acceptable and close to or above 0.95 are considered good fits ( Hu & Bentler, 1999).

**Figure 2.**
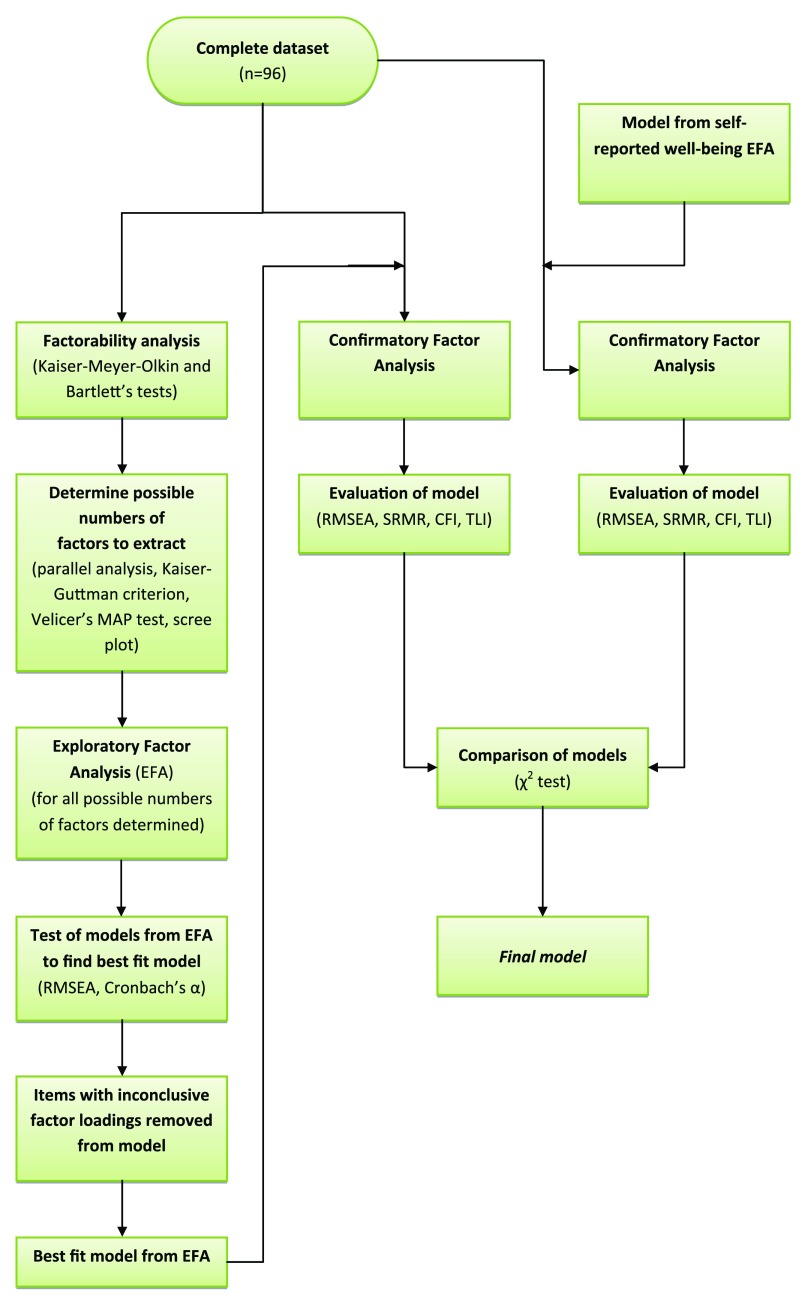
Analysis flowchart for determining best fit model of perceived volunteer well-being factors by volunteer managers.

**Volunteer managers’ perception of volunteers’ well-being:** Only complete responses from former and current volunteer managers were used for factor analysis (n=96) ( Figure 2). Due to the limited sample size, it was not possible to split the data into a development and a test sample, as sample size should be at least 100–200 per subsample to perform the analysis ( MacCallum *et al.*, 1996). EFA was performed on the complete sample, following the method described above, including testing factorability of items, determining number of factors to extract and using oblique (promax) rotation for the EFA. The best fit model was determined also following the described method above by evaluating RMSEA, interpretability and Cronbach’s α. Items with inconclusive factor loadings were removed. CFA was then performed on the volunteer manager data sample using the best-fitting model from the EFA, the model developed from the self-reported well-being sample EFA described above, the original seven-factor PERMA-P model and a one-dimensional control model. Model fit for all models were evaluated using RMSEA, SRMR, CFI and TLI, and models were compared for best fit using χ ^2^ difference tests.

***Influence of volunteering type and other variables on well-being scores.*** As data were non-normally distributed, non-parametric tests were used in all analyses. As samples in the onsite survey (Study 1) had subject replication, Wilcoxon signed-rank tests were used to test for differences in the level of general well-being and level of activity-related well-being within the four groups of activity participants. For all other comparisons without subject replication, Wilcoxon rank sum tests were used to test for differences in levels between general and activity-related well-being. Kruskal-Wallis tests with post hoc Dunn’s test with Bonferroni correction were used to identify significantly different levels of actual and perceived well-being between the four different types of volunteers (Studies 2 and 3) and between managers in the four different types of volunteering (Study 3), respectively.

Stepwise multiple regression was performed to examine if there were any effects of external variables on overall mean well-being, calculated as the mean of all well-being items (23 items) with negative items, i.e. negative emotions and loneliness, reverse scored. Variables included in Study 1 were volunteer frequency, tenure and hours per month volunteered, and specific variables on the day: weather, group size, hours volunteered, volunteer manager experience and type of volunteering. In Study 2, variables included were volunteering type, as well as demographic variables (age, gender, education, country). Variables included in Study 3 were volunteering type, period and manager tenure, as well as demographic variables (age, gender, education, country).

***Statistical analysis.*** All statistical analyses were completed using RStudio v3.2.3 ( RStudio Team, 2015). The nFactor package v.2.3.3 ( Raiche, 2010), psych package v.1.5.8 ( Revelle, 2016) and the GPArotation package v.2014.11-1 ( Bernaards & Jennrich, 2005) were used for exploratory factor analysis, the lavaan package v.0.5-20 for R was used for confirmatory factor analysis ( Rosseel, 2012) and the ggplot2 package v.2.0.0 was used to create graphs ( Wickham, 2009).

## Results

### Studies 1 and 2: Immediate and remembered effects of environmental volunteering, other nature-based activities and other types of volunteering

***Deriving the self-reported well-being factors.*** Factorability of the items in the development sample was supported by a Kaiser-Meyer-Olkin measure of 0.94 and a significant Bartlett’s test of sphericity (χ ^2^(210)=8448.17; p<0.001), indicating the data were fit for factor analysis. The number of factors to extract was determined by evaluating several factor extraction results: parallel analysis suggested six factors, the Kaiser-Guttman criteria suggested four factors, Velicer’s minimum average partial test identified three factors and visual inspection of the scree plot suggested between two and five factors. Three-, four-, five- and six-factor models were evaluated through exploratory factor analysis and Cronbach’s α for individual factors for each model were also evaluated. The five-factor model provided the clearest structure with a good fit (RMSEA = 0.056 [90% confidence interval = 0.048, 0.062]). Five of the seven factors could be interpreted as factors from the PERMA-P ( Table 5): ‘Engagement’ (four items, α = 0.79), ‘relationships’ (three items, α = 0.77), ‘meaning’ (two items, α = 0.88), ‘negative emotions’ (three items, α = 0.64) and ‘health’ (three items, α = 0.92). One ‘positive emotion’ item, ‘In general, how often do you feel joyful?’, loaded on the ‘engagement’ factor. One ‘achievement’ item, ‘How often do you achieve the important goals you have set for yourself?’ loaded on the ‘meaning’ factor, but was dropped to substantially improve internal reliability of factor and overall model fit. Five items failed to load conclusively on any one factor and were left out of the subsequent confirmatory factor analysis.

**Table 5.** Five well-being factors resulting from exploratory factor analysis of the development sample. The five well-being factors resulting from exploratory factor analysis of the development sample. Cronbach's α for each factor and items with factor loadings (only loadings <-0.30 or >0.30). Greyed out items were excluded from the final model due to inconclusive factor loadings, and were not included in the confirmatory factor analysis. One item was dropped to improve internal reliability of factor (n=645).

		Engagement	Relationship	Meaning	Negative	Health
*Cronbach's α*		*0.79*	*0.77*	*0.88*	*0.64*	*0.92*
Item	Original PERMA-P factor					
How often do you become absorbed in what you are doing?	Engagement	**0.84**				
In general, how often do you feel joyful?	Positive emotion	**0.84**				
In general, to what extent do you feel excited and interested in things?	Engagement	**0.65**				
How often do you lose track of time while doing something you enjoy?	Engagement	**0.54**				
In general, how often do you feel positive?	Positive emotion	0.46				
How much of the time do you feel you are making progress towards accomplishing your goals?	Achievement	0.42		0.36		
To what extent do you feel appreciated?	Relationship		**1.06**			
How satisfied are you with your personal relationships?	Relationship		**0.86**			
To what extent do you receive help and support from others when you need it?	Relationship		**0.53**			
In general, to what extent do you feel contented?	Positive emotion		0.47			
To what extent do you generally feel you have a sense of direction in your life?	Meaning		0.40	0.38		
In general, to what extent do you lead a purposeful and meaningful life?	Meaning			**0.99**		
In general, to what extent do you feel that what you do in your life is valuable and worthwhile?	Meaning			**0.69**		
How often do you achieve the important goals you have set for yourself? ^1^	Achievement			0.56		
How often do you feel frustrated?	Negative emotion				**0.66**	
How often do you feel sad?	Negative emotion				**0.63**	
How often do you feel anxious?	Negative emotion				**0.64**	
How satisfied are you with your current physical health?	Health					**0.99**
In general, how would you say your health is?	Health					**0.88**
Compared to others of your same age and sex, how is your health?	Health					**0.89**
How often are you able to handle your responsibilities?	Achievement				

CFA was run on the test sample and the combined development and test sample with the five-factor model developed from the EFA. Model fit was acceptable for the test sample based on all fit indices (RMSEA (0.076 [0.067; 0.085]), SRMR (0.066), CFI (0.938) and TLI (0.918)). Model fit was good for the combined development and test sample based on SRMR (0.055), CFI (0.955) and TLI (0.940) indices and was acceptable based on RMSEA (0.069 [0.064; 0.075]). The five-factor model from the EFA fitted the test sample significantly better than the original seven-factor PERMA-P model (Δχ ^2^(88) = 530; p<0.001) or a generic one-factor model (Δχ ^2^(109) = 1565; p<0.001). The five-factor model also fitted the combined development and test sample significantly better than the original seven-factor PERMA-P model (Δχ ^2^(88) = 788; p<0.001) or a generic one-factor model (Δχ ^2^(109) = 3717; p<0.001). Factor correlations based on the combined test and development sample are summarised in Table 6, and show that all factors were significantly correlated.

**Table 6.** Final well-being factors, descriptive statistics and correlations for the combined development and test participant sample. Final well-being factors, descriptive statistics and correlations for the combined development and test participant sample showing significant correlations between all factors (n=1157; **p<0.001).

Variable	Mean	SD	Engagement	Relationship	Meaning	Negative
Engagement	7.34	1.53	1.00			
Relationship	7.55	1.74	0.52**	1.00		
Meaning	7.73	1.74	0.66**	0.63**	1.00	
Negative	2.77	2.22	-0.20**	-0.45**	-0.39**	1.00
Health	7.47	1.75	0.40**	0.44**	0.50**	-0.35**

***External factors and volunteer well-being.*** Volunteers spending more hours volunteering per month, and for Study 2 also spending more time volunteering outdoors, reported higher levels of overall well-being. For volunteers in Study 1, this result came from stepwise multiple regression, which reduced the model for predicting the overall mean volunteering-related well-being score to only include the number of hours spent volunteering per month as a significant factor (F _1,164_ = 5.55; p<0.05; R ^2^ = 0.03). For the current volunteers in Study 2, stepwise multiple regression reduced the model for predicting the overall mean volunteering-related well-being score to include the number of hours spent volunteering per month (p<0.001) and the amount of time spent outdoors while volunteering (p<0.001) as significant factors (F _2,225_ = 11.69; p<0.001; R ^2^_adj_ = 0.09). The number of hours spent volunteering per month (r=0.22; p<0.001) and the amount of time spent outdoors while volunteering (r=0.21; p<0.01) were both significantly positively correlated with the overall mean volunteering-related well-being score.

**Figure 3.**
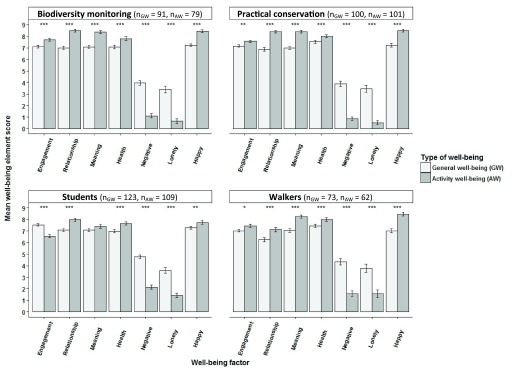
Differences between paired general well-being scores and activity well-being scores of participants in nature-based activities. Differences between paired general well-being scores (light grey) and activity well-being scores (dark grey) for biodiversity monitoring volunteers, practical conservation volunteers, students and walkers (±SE bars). ‘Engagement’, ‘relationship’, ‘meaning’, ‘negative emotion’ and ‘health’ factor scores were means of factor item aggregates. ‘Loneliness’ and ‘happiness’ were single item measures (Wilcoxon signed-rank tests;*p< 0.05, **p<0.01, ***p<0.001).

***Study 1: How does environmental volunteering immediately affect well-being?*** Mean scores were calculated for each well-being element for both general well-being and activity-related well-being in the four participating groups: Biodiversity monitoring volunteers, practical conservation volunteers, students and walkers ( Table 7). All groups rated most of their activity-related well-being significantly better than their general well-being with the positive indices, ‘engagement’, ‘relationship’, ‘meaning’, ‘health’ and ‘happiness’, rated significantly higher and the negative indices, ‘negative emotions’ and ‘loneliness’, rated significantly lower for activity-related well-being than for general well-being (Wilcoxon signed-rank test; p<0.05 for all; Figure 3). The only exceptions were students’ rating of ‘meaning’, which was not significantly different between generally in life and during their fieldwork, and their rating of ‘engagement’, which was significantly lower for activity-related well-being than generally in life.

**Table 7.** Means (SD) for well-being elements for all groups of participants and all types of well-being. BM, biodiversity monitoring volunteers; PC, practical conservation volunteers; BMPC, biodiversity monitoring volunteers also doing practical conservation.

		Study 1 (onsite, paired observations)		Study 2 (online, unpaired observations)		Study 3 (online, managers)
Well-being element	Group	General well-being	Experienced activity-related well-being	General well-being	Remembered volunteer-related well-being	Perceived volunteer well- being
Engagement						
	Students	7.32 (1.12)	6.21 (1.98)			
	Walkers	7.13 (1.29)	7.51 (1.47)			
	BM	7.25 (1.45)	7.83 (1.32)	7.33 (1.56)	7.14 (1.74)	7.50 (1.27)
	PC	7.34 (1.33)	7.69 (1.52)	7.61 (1.33)	7.53 (1.46)	7.73 (1.10)
	BMPC			7.21 (1.59)	7.97 (1.15)	7.64 (1.22)
	Other			7.46 (1.20)	7.61 (1.49)	7.07 (1.85)
Relationship						
	Students	6.88 (1.59)	7.63 (1.50)			
	Walkers	6.36 (1.80)	7.18 (1.87)			
	BM	7.14 (1.58)	8.61 (1.31)	7.11 (2.17)	7.40 (1.64)	7.79 (1.34)
	PC	7.07 (1.75)	8.52 (1.30)	7.11 (2.19)	8.02 (1.35)	8.25 (0.83)
	BMPC			7.49 (1.64)	8.25 (1.59)	8.06 (1.35)
	Other			7.53 (1.78)	8.34 (1.47)	7.89 (1.77)
Meaning						
	Students	6.87 (1.73)	7.06 (2.02)			
	Walkers	7.14 (1.62)	8.31 (1.44)			
	BM	7.20 (1.48)	8.48 (1.27)	7.86 (1.37)	8.07 (1.34)	8.11 (1.08)
	PC	7.18 (1.76)	8.53 (1.58)	7.31 (1.96)	8.18 (1.51)	8.38 (1.04)
	BMPC			7.47 (1.86)	8.55 (1.11)	8.47 (1.25)
	Other			7.72 (1.75)	8.72 (1.45)	8.67 (1.04)
Health						
	Students	6.77 (1.52)	7.31 (1.73)			
	Walkers	7.55 (1.55)	8.06 (1.57)			
	BM	7.19 (1.84)	7.90 (1.89)	6.97 (1.90)	7.37 (1.57)	6.42 (1.63)
	PC	7.72 (1.59)	8.14 (1.52)	7.36 (2.40)	8.00 (1.62)	6.80 (1.81)
	BMPC			7.72 (1.92)	7.81 (1.89)	7.06 (1.80)
	Other			7.10 (1.83)	7.69 (1.92)	5.33 (2.43)
Negative						
	Students	4.55 (1.74)	1.81 (1.74)			
	Walkers	4.43 (2.05)	1.65 (1.71)			
	BM	4.08 (1.80)	1.24 (1.76)	4.17 (2.19)	1.86 (1.66)	2.33 (1.27)
	PC	4.07 (2.10)	1.00 (1.21)	3.75 (1.93)	1.62 (1.36)	2.69 (1.09)
	BMPC			3.94 (2.33)	1.84 (1.78)	2.63 (1.63)
	Other			3.91 (2.26)	2.41 (1.62)	3.72 (2.00)
Lonely						
	Students	3.37 (2.60)	1.07 (2.00)			
	Walkers	3.89 (2.87)	1.63 (2.68)			
	BM	3.54 (2.83)	0.77 (1.88)	3.41 (3.21)	1.11 (1.95)	1.53 (1.82)
	PC	3.66 (3.08)	0.65 (1.41)	2.92 (3.17)	0.96 (1.68)	2.08 (1.60)
	BMPC			3.24 (2.98)	1.17 (2.24)	1.94 (2.20)
	Other			2.41 (2.87)	1.27 (2.04)	1.72 (1.97)
Happy						
	Students	7.06 (1.56)	7.39 (2.10)			
	Walkers	7.12 (1.66)	8.52 (1.48)			
	BM	7.34 (1.50)	8.57 (1.21)	7.32 (2.20)	7.98 (1.67)	7.89 (1.25)
	PC	7.42 (1.75)	8.61 (1.52)	7.62 (1.94)	8.54 (1.29)	8.36 (0.93)
	BMPC			7.47 (2.06)	8.51 (1.46)	8.09 (1.79)
	Other			7.74 (1.73)	8.54 (1.70)	7.50 (2.11)

**Figure 4.**
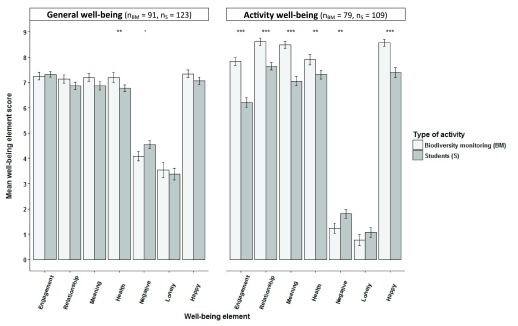
Differences between biodiversity monitoring volunteers and students in their level of general and activity well-being. Differences between biodiversity monitoring volunteers (BM) and students (S) in their level of general well-being (light grey) and activity well-being (dark grey) (±SE bars). ‘Engagement’, ‘relationship’, ‘meaning’, ‘negative emotion’ and ‘health’ factor scores were means of factor item aggregates. ‘Loneliness’ and ‘happiness’ were single item measures (Wilcoxon rank sum test; ·p<0.06, *p<0.05, ***p<0.001).

Comparing biodiversity monitoring volunteers to their student control group for general well-being there was one significant difference, as volunteers rated their ‘health’ significantly higher than students did (Wilcoxon rank sum test; p<0.05; Figure 4). Volunteers also rated their ‘negative emotions’ slightly lower than students did (Wilcoxon rank sum test; p<0.06). When comparing their activity-related well-being, however, there were significant differences in all elements of well-being, except ‘loneliness’, as volunteers consistently rated positive indices significantly higher and ‘negative emotions’ significantly lower than students did (Wilcoxon rank sum tests; p<0.01 for all).

**Figure 5.**
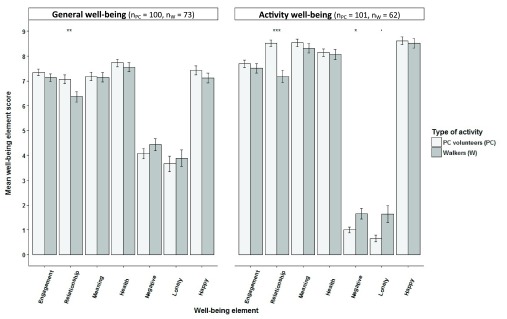
Differences between practical conservation volunteers and walkers in their level of general and activity well-being. Differences between practical conservation volunteers (PC) and walkers (W) in their level of general well-being (light grey) and activity well-being (dark grey) (±SE bars). ‘Engagement’, ‘relationship’, ‘meaning’, ‘negative emotion’ and ‘health’ factor scores were means of factor item aggregates. ‘Loneliness’ and ‘happiness’ were single item measures (Wilcoxon rank sum tests; · p<0.06, *p<0.05, **p<0.01, ***p<0.001).

Comparing practical conservation volunteers to their walker control group for their general level of well-being there was one significant difference, as volunteers rated ‘relationships’ significantly higher than walkers did (Wilcoxon rank sum test; p<0.01; Figure 5). This difference in ‘relationship’ ratings was even more significant when comparing their activity-related well-being (Wilcoxon rank sum test; p<0.001). Also negative indices showed differences in activity-related well-being with volunteers rating their ‘negative emotions’ significantly lower than walkers (Wilcoxon rank sum test; p<0.05) and rating their ‘loneliness’ lower than walkers.

Comparing the two different types of environmental volunteers, the biodiversity monitoring volunteers and the practical conservation volunteers, there were no significant differences in their levels of general (Wilcoxon rank sum tests; p>0.07 for all) or activity-related (Wilcoxon rank sum tests; p>0.30 for all) well-being, suggesting that irrespective of the type of environmental volunteering performed, the effect on well-being is equally positive.

Raw data from study 1, the onsite nature-based activity survey Click here for additional data file. The raw data from onsite questionnaires of environmental volunteers and their control groups (walkers and students) supporting the findings described in the paper are provided. Copyright: © 2016 Kragh G et al. 2016 Data associated with the article are available under the terms of the Creative Commons Zero "No rights reserved" data waiver (CC0 1.0 Public domain dedication).

**Figure 6.**
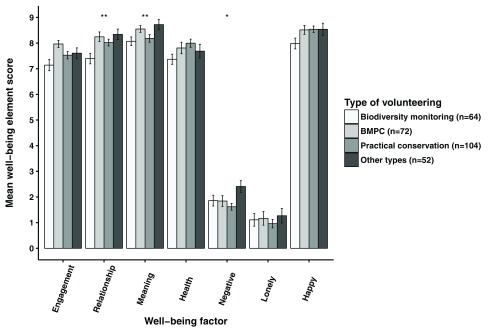
Remembered volunteering-related well-being of different types of current volunteers The remembered volunteering-related well-being of different types of current volunteers (±SE bars) with significant differences found for ‘relationship’, ‘meaning’ and ‘negative emotions’ (Kruskal-Wallis tests; p<0.05, **p<0.01). ‘Engagement’, ‘relationship’, ‘meaning’, ‘negative emotion’ and ‘health’ factor scores were means of factor item aggregates. ‘Loneliness’ and ‘happiness’ were single item measures. BMPC, biodiversity monitoring volunteers also doing practical conservation work.

***Study 2: How well do volunteers sustain the memory of the immediately experienced sense of well-being after they have gone home?*** In the online survey, current volunteers were asked to remember the last time they volunteered and rate how they felt during that time. The ‘relationship’ (Kruskal-Wallis test; χ ^2^(3) = 16.18; p<0.01), ‘meaning’ (Kruskal-Wallis test; χ ^2^(3) = 11.69; p<0.01) and ‘negative emotion’ (Kruskal-Wallis test; χ ^2^(3) = 9.43; p<0.05) elements showed significant differences between different types of volunteers ( Table 7 and Figure 6). Biodiversity monitoring volunteers consistently rated positive indices lower than any other types of volunteers, and significantly so the ‘relationship’ element compared to biodiversity monitoring volunteers also doing practical conservation work (Dunn’s test; z = -3.44; p<0.01) and non-environmental volunteers (Dunn’s test; z = -3.46; p<0.01), and the ‘meaning’ element compared to non-environmental volunteers (Dunn’s test; z = -3.12; p<0.01). Also practical conservation volunteers rated ‘meaning’ significantly lower than non-environmental volunteers (Dunn’s test; z = 2.67; p<0.05). For ‘negative emotions’, however, both practical conservation volunteers (Dunn’s test; z = 2.95; p<0.01) and biodiversity monitoring volunteers also doing practical conservation (Dunn’s test; z = -2.48; p<0.05) rated them significantly lower than non-environmental volunteers.

**Figure 7.**
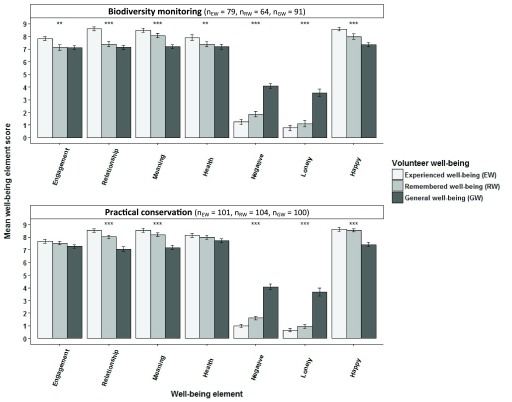
Experienced, remembered and general well-being of environmental volunteers. Experienced well-being just after volunteering ended and remembered volunteering-related well-being up to six months after volunteering compared to volunteers' general level of well-being in life for volunteers in biodiversity monitoring and practical conservation volunteering (±SE bars; Kruskal-Wallis tests; *p<0.05, **p<0.01, ***p<0.001).

Comparison of volunteers’ experienced well-being just after volunteering ended (Study 1), their remembered volunteering-related well-being up to 12 months after volunteering (Study 2) and their general level of well-being in life (paired data from Study 1) showed that biodiversity monitoring volunteers consistently rated experienced positive indices significantly higher than their well-being generally in life (Kruskal-Wallis with post-hoc Dunn’s tests; p<0.01 for all); remembered well-being was rated intermediate and significantly different from immediately experienced well-being for ‘engagement’, ‘relationship’ and ‘health’ (Kruskal-Wallis with post-hoc Dunn’s tests; p<0.01) and significantly different from well-being generally in life for ‘meaning’ and ‘happiness’ (Kruskal-Wallis with post-hoc Dunn’s tests; p<0.01; Table 7; Figure 7). Practical conservation volunteers showed the same trend and also rated their experienced ‘relationship’, ‘meaning’ and ‘happiness’ significantly higher just after volunteering and when later remembering it compared to generally in life (Kruskal-Wallis with post-hoc Dunn’s tests; p<0.001). Both types of volunteers rated ‘negative emotions’ significantly lower just after volunteering and when remembering later than generally in life (Kruskal-Wallis with post-hoc Dunn’s tests; p<0.001 for all).

There was no effect of time since current volunteers last volunteered within the last six months on their well-being ratings (Study 2, n=277; Kruskal-Wallis; p>0.05 for all). Comparing the baseline general well-being of volunteers from Study 1 (n=191) and non-volunteers, defined as people not having volunteered for at least 6 months, from Study 2 (n=51), there were no significant differences in ratings for any well-being elements (Wilcoxon rank sum tests; p>0.05 for all).

Raw data from study 2, the online volunteer survey Click here for additional data file. The raw data from online questionnaires of current, former and potential volunteers supporting the findings described in the paper are provided. Copyright: © 2016 Kragh G et al. 2016 Data associated with the article are available under the terms of the Creative Commons Zero "No rights reserved" data waiver (CC0 1.0 Public domain dedication).

### Study 3: How do volunteer managers perceive the effect of volunteering on the well-being of their volunteers?

***Deriving the perceived well-being factors.*** Exploratory factor analysis performed on the volunteer manager data identified a four-factor model; however, fit indices indicated only marginal fit (RMSEA = 0.09 [90% CI = 0.053; 0.102], TLI = 0.91). CFA based on the four-factor model revealed bad fit (RMSEA = 0.111 [90% CI = 0.089, 0.133], SRMR = 0.097, CFI = 0.880, TLI = 0.850). CFA based on the model from the self-reported well-being sample, but excluding the ‘health’ factor as there was only one item on health in the volunteer manager questionnaire, indicated acceptable fit based on CFI (0.929), TLI (0.902) and SRMR (0.066), though RMSEA (0.100 [90% CI = 0.069, 0.130]) was high. The four-factor model from the self-reported well-being sample had significantly better fit than the model developed from the volunteer manager EFA (Δχ ^2^(36) = 90; p<0.001), the original PERMA-P model (without the ‘health’ factor) (Δχ ^2^(72) = 223, p<0.001) or a generic one-factor model (Δχ ^2^(6) = 146; p<0.001) and it was therefore used for exploring perceived well-being further. Factor correlations based on the volunteer manager sample are summarised in Table 8.

**Table 8.** Final well-being factors, descriptive statistics and correlations for volunteer manager sample. Final well-being factors (‘engagement’, ‘relationship’, ‘meaning’, ‘negative emotion’, 0–10 scale), descriptive statistics and correlations for volunteer manager sample (n=94–96, * p<0.05, **p<0.001). MV Time, manager time spent with volunteers (1–6 scale, 6 being 100%); MPS, mean perceived well-being score from all items; Education, 1–6 scale, 6 being doctorate degree.

Variable	Mean	SD	MV Time	Education	MPS	Engagement	Relationship	Meaning
MV Time	2.66	1.23	1.00					
Education	4.10	1.14	-0.20	1.00				
MPS	7.65	1.01	0.25*	-0.20	1.00			
Engagement	7.53	1.37	0.21*	-0.16	0.81**	1.00		
Relationship	8.02	1.35	0.22*	-0.19	0.86**	0.59**	1.00	
Meaning	8.41	1.15	0.22*	-0.12	0.70**	0.56**	0.67**	1.00
Negative	2.79	1.60	-0.06	0.07	-0.54**	-0.19	-0.37**	-0.08

***External factors and perceived well-being.*** Stepwise multiple regression reduced the model for predicting the overall mean perceived well-being score to only include the significant variable of manager time spent with volunteers (measured on 1–6 scale, 6 being 100%; p<0.05) and the important variable of managers’ level of education (measured on 1–6 scale, 6 being doctoral degree; p<0.07) as important factors (F _2,91_ = 4.93; R ^2^_adj_ = 0.08; p<0.01). Manager time spent with volunteers was significantly positively correlated with the overall mean perceived well-being score, as well as with the perceived ‘engagement’, ‘relationship’ and ‘meaning’ elements ( Table 8).

**Figure 8.**
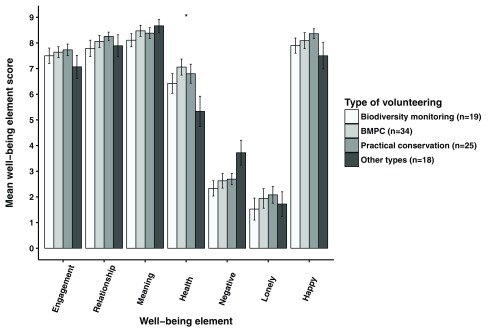
Volunteer managers’ perception of the well-being of their volunteers. The perceived well-being of volunteers by different types of volunteer managers (mean score ±SE bars). Significant difference found only for Health (Kruskal-Wallis test; χ2(3) = 7.63; *p=0.05). ‘Engagement’, ‘relationship’, ‘meaning’ and ‘negative emotion’ factor scores were means of factor item aggregates. ‘Health’, ‘loneliness’ and ‘happiness’ were single item measures. BMPC, biodiversity monitoring volunteers also doing practical conservation work.

***How do volunteer managers perceive the effect of volunteering on the well-being of their volunteers?*** Volunteer managers in different types of volunteering rated the well-being of their volunteers similarly, except for ‘health’ where managers in biodiversity monitoring also doing practical conservation rated their volunteers’ ‘health’ higher than managers in non-environmental volunteering (Dunn’s test; z = 2.69; p<0.05) ( Figure 8).

Raw data from study 3, the online volunteer manager survey Click here for additional data file. The raw data from online questionnaires of current and former volunteer managers supporting the findings described in the paper are provided. Copyright: © 2016 Kragh G et al. 2016 Data associated with the article are available under the terms of the Creative Commons Zero "No rights reserved" data waiver (CC0 1.0 Public domain dedication).

### Studies 1, 2 and 3: How do volunteer manager perceptions of volunteer well-being compare to volunteers’ actual sense of volunteering-related well-being?

**Figure 9.**
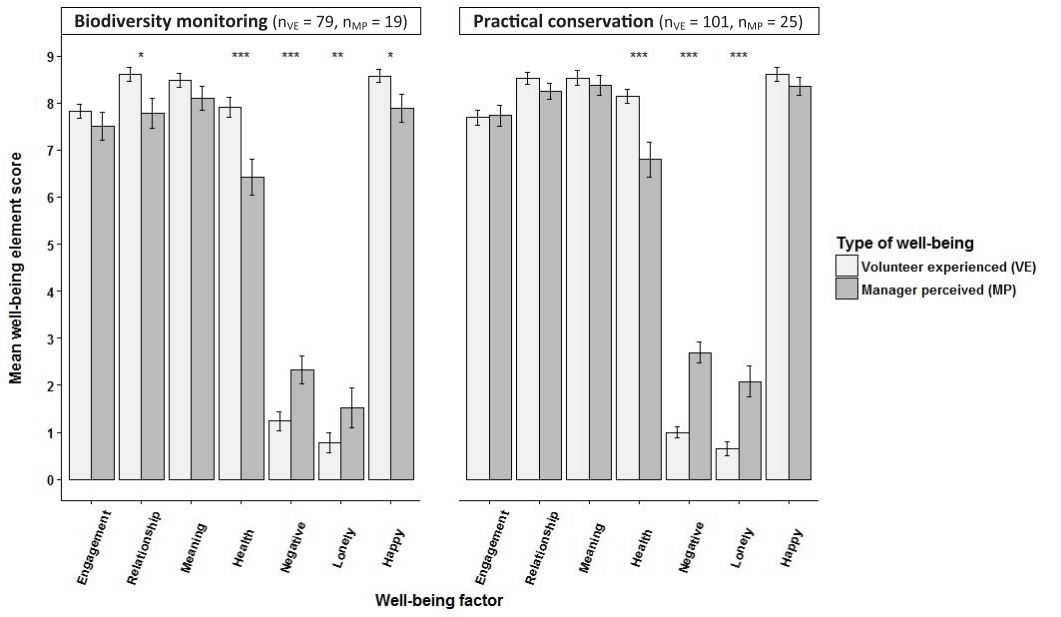
Volunteer experienced well-being compared to volunteer managers’ perception of their volunteers’ well-being. Volunteer experienced well-being just after volunteering ended compared to volunteer managers’ perception of their volunteers’ well-being (±SE bars). ‘Engagement’, ‘relationship’, ‘meaning’, ‘negative emotion’ and ‘health’ factor scores were means of factor item aggregates. ‘Loneliness’ and ‘happiness’ were single item measures. Health was a mean of factor item aggregates for volunteers and a single item for managers (Wilcoxon rank sum tests; *p<0.05, **p<0.01, ***p<0.001).

Volunteer managers’ perception of their volunteers’ well-being corresponded to how volunteers felt just after volunteering ended (‘experienced well-being’) for ‘engagement’ and ‘meaning’ elements of well-being but significantly differed for ‘health’, ‘negative emotions’ and ‘loneliness’ in both biodiversity monitoring and practical conservation volunteering ( Figure 9). Volunteer managers perceived their volunteers as significantly less healthy (Wilcoxon rank sum tests; p<0.001) and as having more ‘negative emotions’ (Wilcoxon rank sum tests; p<0.001) and feeling more ‘lonely’ (Wilcoxon rank sum tests; p<0.01) than was the experience of the volunteers. Managers in biodiversity monitoring also perceived volunteers’ ‘relationship’ and ‘happiness’ elements significantly lower than volunteers reported they felt (Wilcoxon rank sum tests; p<0.05).

**Figure 10.**
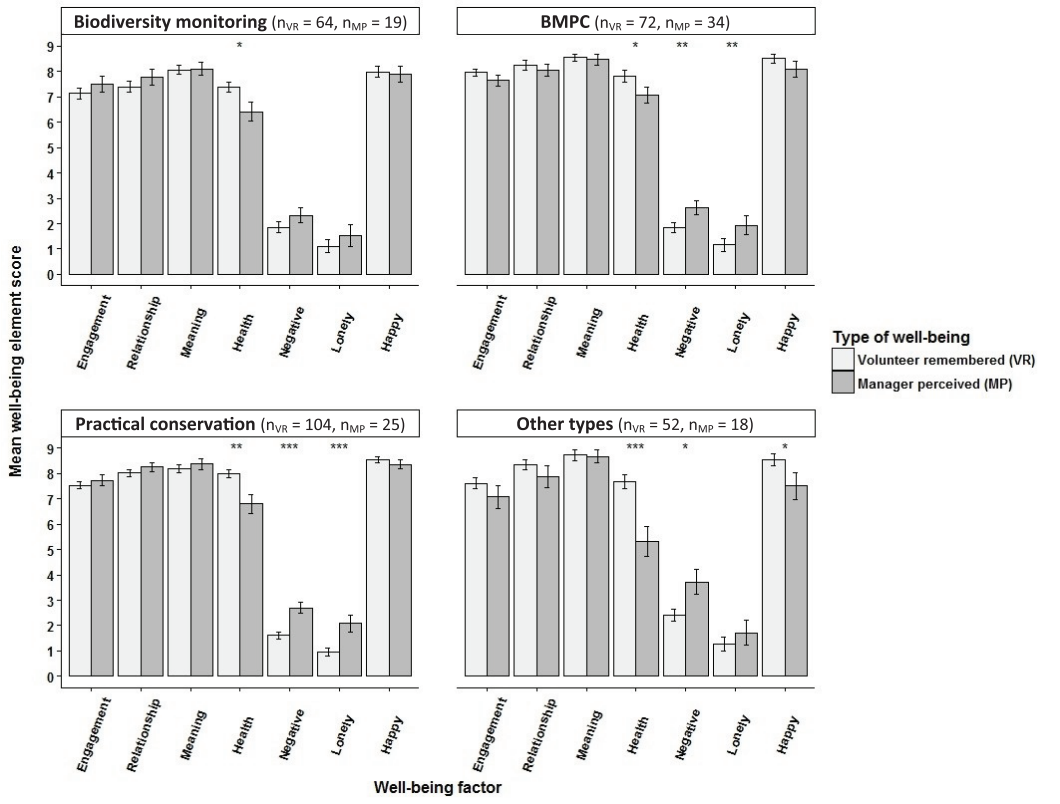
Volunteer remembered well-being compared to volunteer managers’ perception of their volunteers’ well-being. Volunteer remembered well-being compared to volunteer managers’ perception of their volunteers’ well-being (±SE bars). ‘Engagement’, ‘relationship’, ‘meaning’ and ‘negative emotion’ factor scores were means of factor item aggregates. ‘Loneliness’ and ‘happiness’ were single item measures. ‘Health’ was a mean of factor item aggregates for volunteers and a single item for managers (Wilcoxon rank sum tests; *p<0.05, **p<0.01, ***p<0.001). BMPC, biodiversity monitoring volunteers also doing practical conservation work.

When volunteer managers’ perception of the well-being of their volunteers was compared to how volunteers later rated their remembered volunteering-related well-being, there was still a significant difference in all types of volunteering with managers rating their volunteers’ ‘health’ lower than the volunteers (Wilcoxon rank sum tests; p<0.05; Figure 10). Managers rated volunteers’ perceived ‘negative emotions’ significantly higher than volunteers did in all types of volunteering (Wilcoxon rank sum tests; p<0.05), except biodiversity monitoring. Managers also rated volunteers’ perceived ‘loneliness’ significantly higher in both practical conservation and biodiversity monitoring also doing practical conservation volunteering than volunteers (Wilcoxon rank sum tests; p<0.01). In non-environmental volunteering, managers rated volunteers’ perceived ‘happiness’ significantly lower than volunteers (Wilcoxon rank sum test; p<0.05).

## Discussion

Overall, and supporting previous research, volunteering increased participants’ immediate sense of well-being, both by increasing positive elements and by decreasing negative emotions and loneliness, and it did so more than other types of nature-based activities. Remembering the volunteer experience later on, volunteers retained the feeling of a meaningful event with low levels of negative emotions and loneliness, though other positive feelings of engagement or positive relationships were not retained. Contrary to previous research, this study found that volunteering did not increase volunteers’ general level of well-being when compared to non-volunteers’ general level of well-being. Volunteer managers did perceive the increase in the positive elements of their volunteers’ well-being during volunteering but did not perceive the significant decrease in negative emotions and loneliness their volunteers reported. This section will further discuss these points.

### How nature-based activities immediately affects participants’ sense of well-being

All nature-based activities examined in this research had a significant positive effect on some or all elements of participants’ well-being, a result that agrees with previous studies ( Iwata *et al.*, 2016; Koss & Kingsley, 2010; O’Brien *et al.*, 2010; Wyles *et al.*, 2016). However, contrary to many published studies that found volunteers had higher levels of well-being generally in life than non-volunteers (e.g. Greenfield & Marks, 2004; Harlow & Cantor, 1996; Konrath *et al.*, 2012), this study found no significant difference between volunteers and non-volunteers in their general level of well-being. For the online sample in Study 2, reasons for this could be the relatively small sample size for non-volunteers (n=51) and a potential selection bias ( Ahern, 2005) in survey participation, as non-volunteers were not a random sample of people not volunteering, but rather people showing an interest in volunteering, either as former volunteers or potential future volunteers. However, findings in Study 1 were similar to Study 2 though students and walkers did not participate in this survey due to an interest in volunteering, suggesting it was not only a case of selection bias or small sample size.

The finding in the current study that volunteers who spend more time volunteering report higher immediate and remembered well-being supports previous studies ( Binder & Freytag, 2013; Thoits & Hewitt, 2001). One study has suggested that between 100 and 800 volunteer hours per year provided the highest rates of well-being ( Windsor *et al.*, 2008). However, other studies have found that the benefits of volunteering over 100 hours per year either led to no further benefits ( Morrow-Howell *et al.*, 2003) or led to decreased benefits and satisfaction ( Van Willigen, 2000).

The lowered levels of ‘negative emotions’ and ‘loneliness’ during all nature-based activities support previous research showing that volunteering and restorative experiences can decrease mental health issues such as depression ( Korpela *et al.*, 2016; Musick & Wilson, 2008; Pillemer *et al.*, 2010; Townsend, 2006). It also supports the idea that volunteering reduces unhappiness ( Binder & Freytag, 2013; Wilson, 2012), and has a positive effect on the positive elements of people’s well-being.

***Volunteering and physical health.*** Volunteers reported an increase in their health immediately after volunteering, reflecting previous research into practical conservation volunteering where volunteers, even though reporting they were in pain after volunteering, gained a sense of achievement from the pain, and perceived it as something positive ( O’Brien *et al.*, 2010). However, this positive effect did not last as volunteers remembering their health during volunteering later on rated it similar to their general health, which was not different to the health of non-volunteers, suggesting there is no long-term positive effect of volunteering on perceived physical health. This finding supports previous research with similar findings ( Borgonovi, 2008; Jenkinson *et al.*, 2013; Piliavin & Siegl, 2007), though some studies have found a positive relationship between volunteering and physical health ( Pillemer *et al.*, 2010; Thoits & Hewitt, 2001; Van Willigen, 2000).

***Biodiversity monitoring volunteers and students.*** The student group was the only participant group that did not consistently show improvements in all elements of well-being immediately after their activity. The unchanged sense of ‘meaning’ and lowered level of ‘engagement’ among students during their fieldwork could stem from them seeing the fieldwork as a mandatory activity that they did not freely choose, even if they did choose their university course. The feeling of personal control and choice of activity is important for an activity to be seen as a positive experience ( Stukas *et al.*, 1999). As volunteers had freely chosen to participate in their activity, this may be one reason for the differences in activity-related well-being between students and biodiversity monitoring volunteers, even though they were performing the same type of tasks.

***Practical conservation volunteers and walkers.*** Walking has previously been shown to decrease participants’ mental illness and negative affect and increase their sense of well-being (e.g. Iwata *et al.*, 2016; Marselle *et al.*, 2014), which was also found in this study. However, the current research also showed that even bigger decreases in negative affect can be achieved through practical conservation volunteering than through walking, and volunteering can have a positive effect on social relationships as well, an effect not consistently found for walking ( Marselle *et al.*, 2014). The ‘positive relationship’ element included an item on support from others: “To what extent did you receive help and support from others when you needed it during your walk/volunteering today?” This item was particularly differently rated by volunteers and walkers, suggesting that volunteers felt much supported in their volunteering by volunteer managers and other volunteers, whereas walkers possibly either did not perceive a need to be supported or were not supported and therefore rated the item lower than volunteers. For practical conservation volunteers, the coffee and lunch breaks provided additional opportunities for social interactions, which were important to the volunteers, as highlighted by a comment from a practical conservation volunteer to the ‘engagement’ item ‘To what extend did you lose track of time during volunteering today?’

“I never lose track of time, I always know what time it is: It is either before coffee, after coffee, before lunch or after lunch!”

(Male volunteer, Forestry Commission)

Volunteering has previously been found to benefit social well-being ( Koss & Kingsley, 2010; O’Brien *et al.*, 2010; Onyx & Warburton, 2003; Son & Wilson, 2012), which was also the case in this study with practical conservation volunteers having significantly higher levels of ‘positive relationships’, not only during the volunteer activity but also generally in life, than walkers did. Volunteering provides a space where people are having fun with others, can engage in meaningful conversations and feel they are understood, all of which can increase the quality of social relationships ( Reis *et al.*, 2000).

### How volunteers sustained the memory of the experienced sense of well-being

When volunteers recalled their experience of volunteering later on and up to six months after volunteering, their ratings of their well-being during volunteering were less positive than immediately after volunteering. This difference between experienced and remembered well-being during volunteering is likely partly due to recall bias ( Baumeister *et al.*, 2001; Stone *et al.*, 1999), which is the imperfect recollection of past emotions or events by respondents. It has been shown that ‘bad is stronger than good’ ( Baumeister *et al.*, 2001), which means that people remember and put more emphasis on negative events and emotions compared with positive events and emotions. Also volunteers in this research remembered the negative, as in the lowered ‘negative emotions’ and ‘loneliness’, better than the increased positive well-being indices. The ‘meaning’ element retained its high rating over time, supporting previous research that also showed retention of meaning ( Wyles *et al.*, 2016), and suggesting it may be a more robust construct than the ‘engagement’ or ‘relationship’ factors that did not retain their high ratings over time. ‘Meaning’ is part of eudaimonia and as such has been suggested to be longer-lasting than hedonic emotions, or moods, such as ‘positive emotions’ and partly the ‘engagement’ element ( Piliavin, 2009).

### Volunteer managers’ perception of volunteer well-being and how it compares to actual volunteer well-being

Managers in environmental volunteering rated the ‘health’ element of their volunteers’ well-being higher than non-environmental volunteer managers did. This difference between environmental and non-environmental managers’ perception of their volunteers’ health is possibly a reflection of the physical stamina and strength needed to perform environmental volunteering ( O’Brien *et al.*, 2010), whether the tasks are clearing invasive species or walking across uneven ground to record the species composition of an area. Volunteer managers spending more time with their volunteers seemed to better understand the well-being of their volunteers, as they rated their volunteers’ well-being more similar to volunteers’ ratings than managers who spent less time with their volunteers. However, managers still perceived volunteers as having more ‘negative emotions’, being ‘lonelier’ and being in worse ‘health’ than volunteers themselves reported. These worse ratings of negative indices are in line with previous research. A meta-analysis of self-reported and other-reported agreement in well-being ratings found an average correlation of 0.42 between average self-ratings and other-reported ratings for a combined score of life satisfaction, happiness, positive affect and negative affect ( Schneider & Schimmack, 2009). Positive and negative affect measures had relatively low agreement, and negative affect (r=0.18) had less agreement than positive affect (r=0.24) ( Schneider & Schimmack, 2009). Again, this finding could reflect that managers also put more emphasis on and remember negative emotions and events better than positive emotions and events ( Baumeister *et al.*, 2001).

### Using a multidimensional approach to well-being in a volunteering context

It has been suggested that volunteering brings both hedonic and eudaimonic well-being benefits to volunteers ( Piliavin, 2009), and such a multidimensional approach to well-being was supported by this research. It recovered five of the seven proposed factors from the PERMA-P ( Butler & Kern, 2016), including the ‘engagement’, ‘relationship’, ‘meaning’, ‘health’ and ‘negative emotion’ factors, but excluding the ‘positive emotion’ and ‘achievement’ factors. ‘Achievement’ items instead related to both the ‘engagement’ and ‘meaning’ factors, suggesting volunteers may not have set goals for themselves within their volunteering role and therefore not been focused on the achievement of any specific goals. This scenario was also supported by comments from volunteers stating that they did not have specific goals for their volunteering. ‘Positive emotion’ items instead related to the ‘engagement’ and ‘relationship’ factors, suggesting that volunteers did not pursue the positive emotions themselves, but rather that positive emotions arose due to positive relationships and task engagement during volunteering. Future research is needed to further tease apart these relationships in a volunteering context. The value of a multidimensional approach to well-being in the volunteering context is the information gained about how volunteering affects the various elements of well-being differently. In this sample of volunteers, the effects of volunteering were all positive; however, for the students, their engagement decreased during their fieldwork, highlighting an area that should be investigated further to find ways to turn this negative effect around.

### Implications

Walking has been advocated as a public health intervention ( Iwata *et al.*, 2016; Marselle *et al.*, 2014), which the present findings support. However, they also suggest that environmental volunteering may provide increased benefits over and above the benefits of walking. For public health providers, this highlights environmental volunteering as a potential health intervention and a way to reintegrate people into society ( O’Brien *et al.*, 2011) by providing opportunities for positive relationships to develop. However, care must be taken to ensure that people actively choose the activity and do not feel forced to volunteer, as personal control and choice is important for a positive outcome ( Stukas *et al.*, 1999). For volunteer organisations, these positive results highlight that environmental volunteer projects provide benefits to the volunteers themselves and could be useful in motivating people to begin volunteering. In addition, it provides an opportunity to showcase to funding bodies that environmental volunteer projects provide positive outcomes also for the people involved in the projects.

The use of multidimensional well-being measures can provide the information that volunteer organisations and managers need to support and enhance the well-being of their volunteers. By assessing the individual elements, areas for improvement can be specifically targeted. For example, if the ‘meaning’ element is rated low by volunteers, improved feedback could be provided to volunteers to enhance their understanding of their role and thereby the meaning they derive from their volunteering. If ‘relationships’ are rated low, focus should be put on providing adequate support to volunteers during volunteering, as well as ensuring volunteers feel appreciated. Even if volunteers find their roles meaningful and relationships good, their ‘engagement’ may be lacking if they are not given interesting tasks and opportunities to fully immerse themselves in their volunteer tasks.

## Conclusion

This study has shown the benefits of regarding volunteer well-being as a multidimensional construct to better understand how volunteering affects the various elements of well-being. It has highlighted how environmental volunteering immediately improved the well-being of participants, even more than other nature-based activities did. Volunteering improved participants’ well-being especially by lowering negative emotions and loneliness, and this was remembered long after volunteering ended. Most volunteer managers, however, did not perceive this significant decrease in negative emotions and loneliness in their volunteers during volunteering, although they did perceive an increase in positive well-being elements. This focus on negative emotions and events is possibly due to the well-established theory that ‘bad is stronger than good’. Volunteer organisations can use multidimensional assessment of volunteers’ well-being and managers’ perception of their volunteers’ well-being to identify and gain a deeper understanding of actual well-being, gaps in volunteer managers’ perceptions and potential areas for improvement.

## Data availability

The data referenced by this article are under copyright with the following copyright statement: Copyright: © 2016 Kragh G et al.

Data associated with the article are available under the terms of the Creative Commons Zero "No rights reserved" data waiver (CC0 1.0 Public domain dedication). 

**Dataset 1. Raw data from study 1, the onsite nature-based activity survey.**

The raw data from onsite questionnaires of environmental volunteers and their control groups (walkers and students) supporting the findings described in the paper are provided. (DOI: 10.5256/f1000research.10016.d142072; Kragh *et al.*, 2016a).

**Dataset 2. Raw data from study 2, the online volunteer survey.**

The raw data from online questionnaires of current, former and potential volunteers supporting the findings described in the paper are provided. (DOI: 10.5256/f1000research.10016.d142073; Kragh *et al.*, 2016b).

**Dataset 3. Raw data from study 3, the online volunteer manager survey.**

The raw data from online questionnaires of current and former volunteer managers supporting the findings described in the paper are provided. (DOI: 10.5256/f1000research.10016.d142074; Kragh *et al.*, 2016c).

## Supplementary material

**Supplementary Table 1.** Acknowledgements go to all the volunteers, volunteer managers, students and walkers from the organisations detailed in the table.

.

**Supplementary File 1**. The volunteer questionnaires used in Study 1.

Provided here are the before-volunteering and after-volunteering questionnaires used for environmental volunteers. Similar surveys, but with appropriately adapted wording, were used for walkers and students.

.

**Supplementary File 2.** The volunteer questionnaire used in Study 2.

Provided here are the survey items in the online survey pertaining to the volunteer-related well-being of current environmental volunteers. Similar surveys, but with appropriately adapted wording, were used for non-environmental volunteers, as well as former and potential volunteers.

.

**Supplementary File 3.** The volunteer manager questionnaire used in Study 3.

Provided here are the survey items in the online survey pertaining to current environmental volunteer managers’ perception of the well-being of their volunteers. Similar surveys, but with appropriately adapted wording, were used for non-environmental volunteer managers, as well as former volunteer managers.
