# Supplementary material for: Environmental volunteer well-being: Managers’ perception and actual well-being of volunteers
Source: F1000Res. 2016 Nov 16;5:2679. [Version 1] doi: 10.12688/f1000research.10016.1 (PMC5288684; doi:10.12688/f1000research.10016.1)
Supplement: Supplementary file 5 [file f1000research-5-10792-s0004.tgz › df9418fa-9820-4cbd-86aa-299edc6f4eac.pdf]

Bournemouth University

Volunteer Well-being Questionnaire (start-of-day) for PhD project: Enhancing human well-being and wildlife conservation through volunteer engagement

(Please see attached participant information sheet for additional information)

Consent

By completing this questionnaire, I confirm that:

a. I have read and understood the participant information sheet for the above research project and have had the opportunity to ask questions
b. I understand that my participation is voluntary and anonymous and that I am free to withdraw at any time until the data are aggregated and analysed, without giving reason. In addition, should I not wish to answer any particular question(s), I am free to decline
c. I give permission for members of the research team to have access to my anonymised responses
d. I agree to take part in the above research project

Name, position and contact details of researcher:	Name, position and contact details of supervisor:
Gitte Kragh, PhD Candidate, gitte.kragh@bournemouth.ac.uk	Anita Diaz, Associate Professor, adiaz@bournemouth.ac.uk

1. Thinking about your volunteer activities <u>today</u>, how motivated are you about your volunteering <u>today</u> on a scale from 1 (not at all motivated) to 7 (extremely motivated)?

1 (not at all motivated)	2	3	4	5	6	7 (extremely motivated)
☐	☐	☐	☐	☐	☐	☐

- -

2. Please make a code by taking:

**The first 3 letters of the place you were born**	*e.g. London -> write*	***LON***
**The number representing the month you were born**	*e.g. January -> write*	***01***
**The first initial of your mother's first name**	*e.g. Mother's name: Ann -> write*	***A***
	In above example the code is:	***LON01A***

Your code:

_ _

(You will be asked about this code for the afternoon questionnaire again so results can be compared)

Please turn over

3. Please rate the following statements on a scale from 0 to 10

Overall...	Not at all 0	1	2	3	4	5	6	7	8	9	Comple-tely10
How satisfied are you with your life at present?	☐	☐	☐	☐	☐	☐	☐	☐	☐	☐	☐

In general...	Never 0	1	2	3	4	5	6	7	8	9	Always 10
How much of the time do you feel you are making progress towards accomplishing your goals?	☐	☐	☐	☐	☐	☐	☐	☐	☐	☐	☐
How often do you become absorbed in what you are doing?	☐	☐	☐	☐	☐	☐	☐	☐	☐	☐	☐
How often do you feel joyful?	☐	☐	☐	☐	☐	☐	☐	☐	☐	☐	☐
How often do you feel anxious?	☐	☐	☐	☐	☐	☐	☐	☐	☐	☐	☐
How often do you achieve the important goals you have set for yourself?	☐	☐	☐	☐	☐	☐	☐	☐	☐	☐	☐

In general...	Terrible 0	1	2	3	4	5	6	7	8	9	Excellent 10
How would you say your health is?	☐	☐	☐	☐	☐	☐	☐	☐	☐	☐	☐

In general...	Not at all 0	1	2	3	4	5	6	7	8	9	Completely 10
To what extent do you lead a purposeful and meaningful life?	☐	☐	☐	☐	☐	☐	☐	☐	☐	☐	☐
To what extent do you receive help and support from others when you need it?	☐	☐	☐	☐	☐	☐	☐	☐	☐	☐	☐
To what extent do you feel that what you do in your life is valuable and worthwhile?	☐	☐	☐	☐	☐	☐	☐	☐	☐	☐	☐
To what extent do you feel excited and interested in things?	☐	☐	☐	☐	☐	☐	☐	☐	☐	☐	☐
How lonely do you feel in your daily life?	☐	☐	☐	☐	☐	☐	☐	☐	☐	☐	☐

3 (continued). Please rate the following statements on a scale from 0 to 10

	Not at all 0	1	2	3	4	5	6	7	8	9	Completely 10
How satisfied are you with your current physical health?	☐	☐	☐	☐	☐	☐	☐	☐	☐	☐	☐

**In general...**	Never 0	1	2	3	4	5	6	7	8	9	Always 10
How often do you feel positive?	☐	☐	☐	☐	☐	☐	☐	☐	☐	☐	☐
How often do you feel frustrated?	☐	☐	☐	☐	☐	☐	☐	☐	☐	☐	☐
How often are you able to handle your responsibilities?	☐	☐	☐	☐	☐	☐	☐	☐	☐	☐	☐
How often do you feel sad?	☐	☐	☐	☐	☐	☐	☐	☐	☐	☐	☐
How often do you lose track of time while doing something you enjoy?	☐	☐	☐	☐	☐	☐	☐	☐	☐	☐	☐

	Terrible 0	1	2	3	4	5	6	7	8	9	Excellent 10
Compared to others of your same age and sex, how is your health?	☐	☐	☐	☐	☐	☐	☐	☐	☐	☐	☐

**In general...**	Not at all 0	1	2	3	4	5	6	7	8	9	Completely 10
To what extent do you generally feel you have a sense of direction in your life?	☐	☐	☐	☐	☐	☐	☐	☐	☐	☐	☐
How satisfied are you with your personal relationships?	☐	☐	☐	☐	☐	☐	☐	☐	☐	☐	☐
To what extent do you feel appreciated?	☐	☐	☐	☐	☐	☐	☐	☐	☐	☐	☐
To what extent do you feel contented?	☐	☐	☐	☐	☐	☐	☐	☐	☐	☐	☐

	Not at all	1	2	3	4	5	6	7	8	9	Completely
Taking all things together, how happy would you say you are?	☐	☐	☐	☐	☐	☐	☐	☐	☐	☐	☐

Please turn over

**4. How often do you volunteer with monitoring / surveys...**	Several times a week	Once a week	Once or twice a month	Once every 2-3 months	Once or twice a year	Never
**...in groups with *this* organisation?**	☐	☐	☐	☐	☐	☐
**...in groups with *other* organisations?**	☐	☐	☐	☐	☐	☐
**...with friends/family in an informal group?**	☐	☐	☐	☐	☐	☐
**...on your own?**	☐	☐	☐	☐	☐	☐
**This is my first time monitoring with this organisation**	☐					

**5. On average, how many hours per month do you volunteer with monitoring / surveys...**	
**...in groups with *this* organisation?**	________ hours
**...in groups with *other* organisations?**	________ hours
**...with friends/family in an informal group?**	________ hours
**...on your own?**	________ hours

**6 How many years have you volunteered doing monitoring / surveys...**	
**...in groups with *this* organisation?**	________ years
**...in groups with *other* organisations with similar tasks?**	________ years
**...with friends/family in an informal group?**	________ years
**...on your own?**	________ years

**7. How long do you intend to keep volunteering with monitoring / surveys for?**	
Less than a year	☐
1-2 years	☐
3 years or more	☐

Please go to conservation question page and fill in the morning part

Researcher use only

A. Date: ______________

B. Volunteering type:

☐ Monitoring

☐ Other: ___________________________

C. Data entered: ____

Volunteer Well-being Questionnaire (end-of-day) for PhD project:
Enhancing human well-being and wildlife conservation
through volunteer engagement
(Please see attached participant information sheet for additional information)

Consent

By completing this questionnaire, I confirm that:

a. I have read and understood the participant information sheet for the above research project and have had the opportunity to ask questions
b. I understand that my participation is voluntary and anonymous and that I am free to withdraw at any time until the data are aggregated and analysed, without giving reason. In addition, should I not wish to answer any particular question(s), I am free to decline
c. I give permission for members of the research team to have access to my anonymised responses
d. I agree to take part in the above research project

Name, position and contact details of researcher:

Gitte Kragh, PhD student, gitte.kragh@bournemouth.ac.uk

Name, position and contact details of supervisor:

Anita Diaz, Senior Lecturer, adiaz@bournemouth.ac.uk

1. <u>In general</u>, how motivated are you about your volunteering on a scale from 1 (not at all motivated) to 7 (extremely motivated)?

1 (not at all motivated)	2	3	4	5	6	7 (extremely motivated)
☐	☐	☐	☐	☐	☐	☐

2. How many hours did you volunteer today: _ _ _ _ _ _ _ _

3. Please make a code by taking:

**The first 3 letters of the place you were born**	*e.g. London -> write*	***LON***
**The number representing the month you were born**	*e.g. January -> write*	***01***
**The first initial of your mother's first name**	*e.g. Mother's name: Ann -> write*	***A***
	In above example the code is:	***LON01A***

Your code: _

(Same code as this morning)

Please turn over

4. Please rate the following statements on a scale from 0 to 10

Overall...	Not at all 0	1	2	3	4	5	6	7	8	9	Completely 10
How satisfied are you with your volunteering experience today?	☐	☐	☐	☐	☐	☐	☐	☐	☐	☐	☐

	Never 0	1	2	3	4	5	6	7	8	9	Always 10
How much of the time did you feel you were making progress towards accomplishing your goals volunteering today?	☐	☐	☐	☐	☐	☐	☐	☐	☐	☐	☐
To what extent did you become absorbed in your volunteering tasks today?	☐	☐	☐	☐	☐	☐	☐	☐	☐	☐	☐
How much of the time did you feel joyful during volunteering today?	☐	☐	☐	☐	☐	☐	☐	☐	☐	☐	☐
How much of the time did you feel anxious during your volunteering today?	☐	☐	☐	☐	☐	☐	☐	☐	☐	☐	☐

	Terrible 0	1	2	3	4	5	6	7	8	9	Excellent 10
How would you say your health is right now after volunteering?	☐	☐	☐	☐	☐	☐	☐	☐	☐	☐	☐

	Not at all 0	1	2	3	4	5	6	7	8	9	Completely 10
To what extent did you achieve the important goals you had set for your-self during your volunteering today?	☐	☐	☐	☐	☐	☐	☐	☐	☐	☐	☐
To what extent did you find your volunteering today purposeful and meaningful?	☐	☐	☐	☐	☐	☐	☐	☐	☐	☐	☐
To what extent did you receive help and support from others when you needed it during your volunteering today?	☐	☐	☐	☐	☐	☐	☐	☐	☐	☐	☐
To what extent did you feel that what you did during your volunteering today was valuable and worthwhile?	☐	☐	☐	☐	☐	☐	☐	☐	☐	☐	☐
To what extent did you feel excited and interested in things during your volunteering today?	☐	☐	☐	☐	☐	☐	☐	☐	☐	☐	☐
How lonely did you feel during your volunteering today?	☐	☐	☐	☐	☐	☐	☐	☐	☐	☐	☐

4 (continued). Please rate the following statements on a scale from 0 to 10

	Not at all 0	1	2	3	4	5	6	7	8	9	Completely 10
How satisfied are you with your physical health right now after having volunteered?	☐	☐	☐	☐	☐	☐	☐	☐	☐	☐	☐

	Never 0	1	2	3	4	5	6	7	8	9	Always 10
How much of the time did you feel positive during volunteering today?	☐	☐	☐	☐	☐	☐	☐	☐	☐	☐	☐
How much of the time did you feel frustrated during volunteering today?	☐	☐	☐	☐	☐	☐	☐	☐	☐	☐	☐
During your volunteering today, how often were you able to handle your responsibilities?	☐	☐	☐	☐	☐	☐	☐	☐	☐	☐	☐
How much of the time did you feel sad during volunteering today?	☐	☐	☐	☐	☐	☐	☐	☐	☐	☐	☐
To what extent did you lose track of time during volunteering today?	☐	☐	☐	☐	☐	☐	☐	☐	☐	☐	☐

	Terrible 0	1	2	3	4	5	6	7	8	9	Excellent 10
Compared to others of your same age and sex, how is your health right now after volunteering?	☐	☐	☐	☐	☐	☐	☐	☐	☐	☐	☐

	Not at all 0	1	2	3	4	5	6	7	8	9	Completely 10
To what extent do you feel you have a sense of direction in your volunteering?	☐	☐	☐	☐	☐	☐	☐	☐	☐	☐	☐
How satisfied were you with your interactions with other people during volunteering today?	☐	☐	☐	☐	☐	☐	☐	☐	☐	☐	☐
To what extent have you been feeling appreciated during your volunteering today?	☐	☐	☐	☐	☐	☐	☐	☐	☐	☐	☐
To what extent do you feel contented right now after having volunteered?	☐	☐	☐	☐	☐	☐	☐	☐	☐	☐	☐

	Not at all 0	1	2	3	4	5	6	7	8	9	Completely 10
How happy are you right now after volunteering?	☐	☐	☐	☐	☐	☐	☐	☐	☐	☐	☐

Please turn over

Please write any comments you have about this questionnaire and the questions:

Please write any comments you have about what well-being means to you:

Please return to your team leader or Gitte

Thank you for your help!

Researcher use only

A. Date: _______________

C. Data entered: ____

B. Volunteering type:

☐ Monitoring

☐ Conservation tasks

☐ Other: ______________________________
