# Supplementary material for: Environmental volunteer well-being: Managers’ perception and actual well-being of volunteers
Source: F1000Res. 2016 Nov 16;5:2679. [Version 1] doi: 10.12688/f1000research.10016.1 (PMC5288684; doi:10.12688/f1000research.10016.1)
Supplement: Supplementary file 6 [file f1000research-5-10792-s0005.tgz › f46854ce-d1f5-48ac-96f3-b4aecf833924.pdf]

Supplementary material 2. Items from the environmental volunteer questionnaire used in Study 2, the online survey, pertaining to the volunteer-related well-being of current volunteers as well as demographic questions. Similar surveys, but with appropriately adapted wording, were used for non-environmental volunteers as well as former and potential volunteers.

Survey about volunteering

We would like to start off by asking you some general questions.

What is your gender?

- Female
- Male

Please tick the highest completed level of your educational qualifications

Choose one of the following answers

- Left school at 16 (e.g. GCSE/O levels)
- Left school at 18 (e.g., A levels)
- Trade/technical/vocational qualification
- First degree (Bachelor)
- Master’s degree
- Doctoral degree

What is your current employment?
Choose one of the following answers

- Retired
- Full-time employment
- Part-time employment
- Not currently employed
- Homemaker
- Student
- Other

What is your age?
Only numbers may be entered in this field.

Which country do you currently live in?
Choose one of the following answers

- United Kingdom
- United States
- Australia
- Other country

Which environmental or conservation organisation or entity have you volunteered *most often* (most times) with in the last 12 months *(e.g. RSPB, a local wildlife trust, BTO, etc)*?

For example, if you volunteer for two organisations, one whole day once a week with organisation 1 and 2 hours twice a week with organisation 2, please write the name of organisation 2

Please answer all further questions about your volunteering for this particular 'main' organisation, unless otherwise stated.

The next set of questions explores volunteer well-being. Volunteer well-being is important and therefore even though some of the following questions might seem slightly odd, please reply as best you can. The questions are in relation to your well-being whilst you volunteer. There are 4 pages with questions about your well-being whilst volunteering.

Please rate the following statements on a scale from 0 to 10

	0 (not at all)	1	2	3	4	5	6	7	8	9	10 (completely)
Overall, how satisfied are you with your volunteer experience?	0 (not at all)	1	2	3	4	5	6	7	8	9	10 (completely)

Please rate the following statements on a scale from 0 to 10

	0 (never)	1	2	3	4	5	6	7	8	9	10 (always)
How much of the time do you feel you are making progress towards accomplishing your goals while volunteering?	0 (never)	1	2	3	4	5	6	7	8	9	10 (always)
To what extent do you become absorbed in your volunteering tasks?	0 (never)	1	2	3	4	5	6	7	8	9	10 (always)
How much of the time do you feel joyful during volunteering?	0 (never)	1	2	3	4	5	6	7	8	9	10 (always)
How much of the time do you feel anxious during your volunteering?	0 (never)	1	2	3	4	5	6	7	8	9	10 (always)

Your sense of well-being whilst volunteering (continued).

Please rate the following statements on a scale from 0 to 10

	0 (terrible)	1	2	3	4	5	6	7	8	9	10 (excellent)
How would you say your health is right after volunteering?	0 (terrible)	1	2	3	4	5	6	7	8	9	10 (excellent)

Please rate the following statements on a scale from 0 to 10

	0 (not at all)	1	2	3	4	5	6	7	8	9	10 (completely)
To what extent do you achieve the important goals you set for yourself during your volunteering?	0 (not at all)	1	2	3	4	5	6	7	8	9	10 (completely)
To what extent do you find your volunteering purposeful and meaningful?	0 (not at all)	1	2	3	4	5	6	7	8	9	10 (completely)
To what extent do you receive help and support from others when you need it during your volunteering?	0 (not at all)	1	2	3	4	5	6	7	8	9	10 (completely)
To what extent do you feel that what you do during your volunteering is valuable and worthwhile?	0 (not at all)	1	2	3	4	5	6	7	8	9	10 (completely)
To what extent do you feel excited and interested in things during your volunteering?	0 (not at all)	1	2	3	4	5	6	7	8	9	10 (completely)
How lonely do you feel during your volunteering?	0 (not at all)	1	2	3	4	5	6	7	8	9	10 (completely)

Your sense of well-being whilst volunteering (continued).

Please rate the following statements on a scale from 0 to 10

	0 (not at all)	1	2	3	4	5	6	7	8	9	10 (completely)
How satisfied are you with your physical health right after having volunteered?	0 (not at all)	1	2	3	4	5	6	7	8	9	10 (completely)

Please rate the following statements on a scale from 0 to 10

	0 (never)	1	2	3	4	5	6	7	8	9	10 (always)
How much of the time do you feel positive during volunteering?	0 (never)	1	2	3	4	5	6	7	8	9	10 (always)
How much of the time do you feel frustrated during volunteering?	0 (never)	1	2	3	4	5	6	7	8	9	10 (always)
During your volunteering, how often are you able to handle your responsibilities?	0 (never)	1	2	3	4	5	6	7	8	9	10 (always)
How much of the time do you feel sad during volunteering?	0 (never)	1	2	3	4	5	6	7	8	9	10 (always)
To what extent do you lose track of time during volunteering?	0 (never)	1	2	3	4	5	6	7	8	9	10 (always)

Your sense of well-being whilst volunteering (continued). These are the last questions related to your sense of well-being whilst volunteering.

Please rate the following statements on a scale from 0 to 10

	0 (terrible)	1	2	3	4	5	6	7	8	9	10 (excellent)
Compared to others of your same age and sex, how is your health right after volunteering?	○ **0 (terrible)**	○ **1**	○ **2**	○ **3**	○ **4**	○ **5**	○ **6**	○ **7**	○ **8**	○ **9**	○ **10 (excellent)**

Please rate the following statement on a scale from 0 to 10

	0 (not at all)	1	2	3	4	5	6	7	8	9	10 (completely)
To what extent do you feel you have a sense of direction in your volunteering?	○ **0 (not at all)**	○ **1**	○ **2**	○ **3**	○ **4**	○ **5**	○ **6**	○ **7**	○ **8**	○ **9**	○ **10 (completely)**
How satisfied are you with your interactions with other people during volunteering?	○ **0 (not at all)**	○ **1**	○ **2**	○ **3**	○ **4**	○ **5**	○ **6**	○ **7**	○ **8**	○ **9**	○ **10 (completely)**
To what extent do you feel appreciated during your volunteering?	○ **0 (not at all)**	○ **1**	○ **2**	○ **3**	○ **4**	○ **5**	○ **6**	○ **7**	○ **8**	○ **9**	○ **10 (completely)**
To what extent do you feel contented right after having volunteered?	○ **0 (not at all)**	○ **1**	○ **2**	○ **3**	○ **4**	○ **5**	○ **6**	○ **7**	○ **8**	○ **9**	○ **10 (completely)**
How happy are you right after volunteering?	○ **0 (not at all)**	○ **1**	○ **2**	○ **3**	○ **4**	○ **5**	○ **6**	○ **7**	○ **8**	○ **9**	○ **10 (completely)**
