# Supplementary material for: Environmental volunteer well-being: Managers’ perception and actual well-being of volunteers
Source: F1000Res. 2016 Nov 16;5:2679. [Version 1] doi: 10.12688/f1000research.10016.1 (PMC5288684; doi:10.12688/f1000research.10016.1)
Supplement: Supplementary file 7 [file f1000research-5-10792-s0006.tgz › ab824a59-49d9-4120-bb49-c26482f536ad.pdf]

Supplementary material 3. Items from the environmental volunteer manager questionnaire used in Study 3, the online survey, pertaining to the managers' perception of volunteer well-being as well as demographic items. Similar surveys, but with appropriately adapted wording, were used for non-environmental volunteer managers and former volunteer managers.

Survey for volunteer managers / coordinators / leaders

We would like to start off by asking you some general questions.

What is your gender?

- Female
- Male

Please tick the highest completed level of your educational qualifications

- Left school at 16 (e.g. GCSE/O levels)
- Left school at 18 (e.g., A levels)
- Trade/technical/vocational qualification
- First degree (Bachelor)
- Master's degree
- Doctoral degree

What is your current employment?

- Retired
- Full-time employment
- Part-time employment
- Not currently employed
- Homemaker
- Student
- Other

What is your age?

Only numbers may be entered in this field.

Which country do you currently live in?

- United Kingdom
- United States
- Australia
- Other country

Which environmental or conservation organisation or entity have you managed volunteers for *most often* (most times) in the last 12 months *(e.g. RSPB, a local wildlife trust, BTO, etc)*?

(For example, if you manage volunteers for two organisations, one whole day once a week with organisation 1 and 2 hours twice a week with organisation 2, please write the name of organisation 2)

Please answer all further questions in relation to your volunteer management for this particular 'main' organisation.

In total, how many years have you managed volunteers...

Only numbers may be entered in these fields.

...for your 'main' organisation?

...for any other organisation?

The next set of questions explores perceptions of volunteer well-being. The questions are in relation to your perception of the well-being of volunteers whilst they volunteer.

(There are 4 pages with questions about volunteer well-being whilst volunteering)

Please rate the following statement related to your perception of volunteer well-being in your 'main' organisation.

To what extent do you think that volunteers...

	0 (not at all)	1	2	3	4	5	6	7	8	9	10 (completely)
...are satisfied with their volunteer experience overall?	○ **0 (not at all)**	○ **1**	○ **2**	○ **3**	○ **4**	○ **5**	○ **6**	○ **7**	○ **8**	○ **9**	○ **10 (completely)**

Please rate the following statements related to your perception of volunteer well-being in your 'main' organisation.

To what extent do you think that volunteers...

	0 (never)	1	2	3	4	5	6	7	8	9	10 (always)
...feel they are making progress towards accomplishing their goals while volunteering?	○ **0 (never)**	○ **1**	○ **2**	○ **3**	○ **4**	○ **5**	○ **6**	○ **7**	○ **8**	○ **9**	○ **10 (always)**
...become absorbed in their volunteering tasks?	○ **0 (never)**	○ **1**	○ **2**	○ **3**	○ **4**	○ **5**	○ **6**	○ **7**	○ **8**	○ **9**	○ **10 (always)**
...feel joyful during their volunteering?	○ **0 (never)**	○ **1**	○ **2**	○ **3**	○ **4**	○ **5**	○ **6**	○ **7**	○ **8**	○ **9**	○ **10 (always)**
...feel anxious during their volunteering?	○ **0 (never)**	○ **1**	○ **2**	○ **3**	○ **4**	○ **5**	○ **6**	○ **7**	○ **8**	○ **9**	○ **10 (always)**

Volunteers' sense of well-being whilst volunteering (continued).

Please rate the following statements related to your perception of volunteer well-being in your 'main' organisation.

To what extent do you think that volunteers...

	0 (not at all)	1	2	3	4	5	6	7	8	9	10 (completely)
...achieve the important goals they set for themselves during their volunteering?	0 (not at all)	1	2	3	4	5	6	7	8	9	10 (completely)
...find their volunteering purposeful and meaningful?	0 (not at all)	1	2	3	4	5	6	7	8	9	10 (completely)
...feel they receive help and support from others when they need it during their volunteering?	0 (not at all)	1	2	3	4	5	6	7	8	9	10 (completely)
...feel that what they do during their volunteering is valuable and worthwhile?	0 (not at all)	1	2	3	4	5	6	7	8	9	10 (completely)
...feel excited and interested in things during their volunteering?	0 (not at all)	1	2	3	4	5	6	7	8	9	10 (completely)
...feel lonely during their volunteering?	0 (not at all)	1	2	3	4	5	6	7	8	9	10 (completely)

Volunteers' sense of well-being whilst volunteering (continued).

Please rate the following statement related to your perception of volunteer well-being in your 'main' organisation.

To what extent do you think that volunteers...

	0 (not at all)	1	2	3	4	5	6	7	8	9	10 (completely)
...are satisfied with their physical health right after having volunteered?	0 (not at all)	1	2	3	4	5	6	7	8	9	10 (completely)

Please rate the following statements related to your perception of volunteer well-being in your 'main' organisation.

To what extent do you think that volunteers...

	0 (never)	1	2	3	4	5	6	7	8	9	10 (always)
...feel positive during volunteering?	0 (never)	1	2	3	4	5	6	7	8	9	10 (always)
...feel frustrated during volunteering?	0 (never)	1	2	3	4	5	6	7	8	9	10 (always)
...are able to handle their responsibilities during their volunteering?	0 (never)	1	2	3	4	5	6	7	8	9	10 (always)
...feel sad during volunteering?	0 (never)	1	2	3	4	5	6	7	8	9	10 (always)
...lose track of time during volunteering?	0 (never)	1	2	3	4	5	6	7	8	9	10 (always)

Volunteers' sense of well-being whilst volunteering (continued).

Please rate the following statements related to your perception of volunteer well-being in your 'main' organisation.

To what extent do you think that volunteers...

	0 (not at all)	1	2	3	4	5	6	7	8	9	10 (completely)
...feel they have a sense of direction in their volunteering?	**0 (not at all)**	**1**	**2**	**3**	**4**	**5**	**6**	**7**	**8**	**9**	**10 (completely)**
...are satisfied with their interactions with other people during volunteering?	**0 (not at all)**	**1**	**2**	**3**	**4**	**5**	**6**	**7**	**8**	**9**	**10 (completely)**
...feel appreciated during their volunteering?	**0 (not at all)**	**1**	**2**	**3**	**4**	**5**	**6**	**7**	**8**	**9**	**10 (completely)**
...feel contented right after having volunteered?	**0 (not at all)**	**1**	**2**	**3**	**4**	**5**	**6**	**7**	**8**	**9**	**10 (completely)**
...are happy right after volunteering?	**0 (not at all)**	**1**	**2**	**3**	**4**	**5**	**6**	**7**	**8**	**9**	**10 (completely)**
