# Supplementary material for: Environmental volunteer well-being: Managers’ perception and actual well-being of volunteers
Source: F1000Res. 2016 Nov 16;5:2679. [Version 1] doi: 10.12688/f1000research.10016.1 (PMC5288684; doi:10.12688/f1000research.10016.1)
Supplement: Peer review history

# Peer review: Referee response for version 1

**DOI:** 10.5256/f1000research.10792.r19742

**Published:** 2017-01-30

**Authors:** Sabine Pahl

**Affiliations:** 1 School of Psychology, Plymouth University, Plymouth, UK

Title and Abstract:

 This is fine. I have some comments on the comparisons and causality below that the authors should consider.

 Article content/ Conclusions:

 The article is well written and overall clearly structured. Using the PERMA model is a good addition. The specific research questions are very helpful in communicating the research. Nevertheless I have picked up two issues that are worth considering, and a few minor comments.

 I wasn’t entirely convinced by the research question looking at managers’ perception of volunteer well-being. Why is it important that these correspond (p. 3)? Even if it is important, as far as I understand, the comparison is not straightforward. The volunteers rated by the managers are not the same people as rate their own well-being, are they? So the conclusion of non-correspondence is problematic, if you’re comparing the rated/perceived well-being of *different* people.

 My second issue is around the language/interpretation in the article, specifically in the Implications section. You seem to assume these are causal effects i.e. the volunteering causes people’s improved well-being (and therefore it should be used more widely). But it’s not quite that straightforward, as you haven’t allocated people to different activities so there might be other differences between people who walk vs. volunteer for example, that could account for any effects you find. You can only make strong inferences about causality when you use a proper experimental research design. It would be good to note this in the discussion. (I think only the Wyles et al. article has tried this in the volunteering literature). You mention also that choice is important, which is a related consideration. This is where recommendations are a bit tricky, because you can’t (by definition) force people to ‘volunteer’ even it is good for them, and there may be selection effects that mean happier / healthier people are also the ones who do environmental volunteering. This is not a big problem but I feel should be acknowledged.

 Minor points:

- I think a lot of space is dedicated to the different factor analyses (on pages 7-11) to establish questionnaire structure. While this is important and good practice it is not linked to any of the main research questions. Therefore I was wondering if (some of) this should be presented in an Appendix rather than the main text, as it distracts from the key questions and findings.
- On p. 18 literature on the amount of time spent volunteering is reviewed but this all seems to be published in gerontology journals so I’m assuming uses older samples. Please add in the text if that’s the case.

Data:

 Links to raw data are provided.

I have read this submission. I believe that I have an appropriate level of expertise to confirm that it is of an acceptable scientific standard.

# Peer review: Referee response for version 1

**DOI:** 10.5256/f1000research.10792.r17678

**Published:** 2016-11-29

**Authors:** Sarah Elizabeth West

**Affiliations:** 1 Stockholm Environment Institute at York, University of York, York, UK

The abstract results section could be clearer, in particular the sentence starting ‘ Even remembering’.

 I think it would be useful in the introduction to give the geographical context for your work, and figures about the size of the environmental volunteering sector in that country. I assumed UK, and it seems like the bulk of responses were from the UK, but I note that your survey was completed by people in 11 countries. It also needs some definition of environmental volunteering I think. I guess this includes things like practical conservation, environmental CS surveys, but what about someone delivering leaflets promoting Friends of the Earth activities for example? This example highlights why definition is important. And in your results, you talk about Biodiversity monitoring volunteers – is this your definition of environmental volunteers?

 Some justification of why PERMA was used as opposed to other multidimensional well-being measures would be useful.

 Some more info on why managers’ perceptions of their volunteers’ motivations is important is needed, I think this is missing.

 ‘Worldwide responses’ – how do you know that any difference in responses is due to the factors you are interested in, not due to the fact that they are in a different part of the world? Some justification for including these (relatively small number of responses) would be useful.

 The results text is very dense, and it is hard for those not very familiar with factor analysis (like me!) to understand what the key parts of the text are. I guess it’s the bottom of page 9 is it? I think some explanatory text at the beginning of results about what factor analysis is would be helpful. The 'External factors and volunteer well-being' section is clearer as you’ve said what the results are and then gone into the detail of how you came to that result, and means that people who are not au fait with statistics (as I guess will be many of your readers) can skip over it.

 Discussion – how did your volunteers and non volunteers compare to others using your well-being index? Or compared to other well-being indices? This would help to give your results more context.

 Some of your sentences are a little long which makes them a bit hard to read, for example, the one starting However, this positive…on page 19.

 Should your figures be in the discussion section, or would they be better placed in the results? It breaks the text up a bit too much I feel.

I have read this submission. I believe that I have an appropriate level of expertise to confirm that it is of an acceptable scientific standard.
